# Securing Federated Learning With Blockchain in the Medical Field: Systematic Literature Review

**DOI:** 10.2196/79052

**Published:** 2026-02-19

**Authors:** Xudong Wang, Yi Xie, Xiaoliang Chen, Jiaming Yang, Ruiyuan Li, Weihang Gao, Zineng Yan, Hong Zhou, Zhewei Ye

**Affiliations:** 1 Department of Orthopedics, Union Hospital, Tongji Medical College, Huazhong University of Science and Technology, 1277 Jiefang Avenue, Wuhan, Hubei Province, 430022, China, 86 1-397-121-3880; 2 Renmin Hospital of Wuhan University, Wuhan, Hubei Province, China; 3 Department of Orthopedics, People's Hospital of Ningxia Hui Autonomous Region, Ningxia Medical University, Yinchuan, China

**Keywords:** blockchain, federated learning, health care, health data, review, Internet of Medical Things, COVID-19, IoMT

## Abstract

**Background:**

The exponential growth of medical data and advancements in artificial intelligence (AI) have accelerated the development of data-driven health care. However, the secure and efficient sharing of sensitive medical data across institutions remains a major challenge due to privacy concerns, data silos, and regulatory restrictions. Traditional centralized systems are prone to data breaches and single points of failure, while existing privacy-preserving techniques face high computational and communication costs.

**Objective:**

This study aims to provide a comprehensive review of the recent advances in blockchain-based federated learning (BCFL) within the medical field. By exploring the synergistic integration of federated learning and blockchain, this review evaluates how BCFL enhances data security, supports privacy-preserving cross-institutional collaboration, and facilitates practical applications in health care, including medical data sharing, Internet of Medical Things, public health surveillance, and telemedicine.

**Methods:**

We conducted a systematic literature review using databases such as PubMed, IEEE Xplore, Web of Science, and Google Scholar. Boolean logic and domain-specific keywords were used to retrieve studies from 2018 to 2025. After automated deduplication and multistage manual screening, over 100 high-quality papers were included. These works cover BCFL’s theoretical foundations, system architectures, application domains, limitations, and future directions.

**Results:**

BCFL frameworks combine the decentralized trust and auditability of blockchain with the privacy-preserving collaborative learning capabilities of federated learning. This integration mitigates risks such as model tampering, data leakage, and a lack of incentives in federated systems. Applications span across cross-institutional medical data sharing, Internet of Medical Things, epidemic forecasting, and telemedicine. Architectures including fully coupled, flexibly coupled, and loosely coupled models offer varying trade-offs between efficiency, scalability, and security.

**Conclusions:**

BCFL represents a transformative paradigm for secure, collaborative, and privacy-preserving medical AI. By combining decentralized trust, incentive-driven participation, and privacy-enhancing machine learning, BCFL paves the way for next-generation smart health care systems. Despite current technical and practical challenges, BCFL demonstrates strong potential to support precision medicine, global health data collaboration, and large-scale AI deployment in health care.

## Introduction

### Background

With the continuous advancement of medical informatization, the volume of medical big data is growing exponentially, offering significant opportunities for the application of artificial intelligence (AI) in health care, particularly in areas such as assisted diagnosis, personalized treatment, and disease prediction. Especially in recent years, advances in computing power and algorithmic innovation have made machine learning (ML) models a cornerstone of medical intelligence, with their efficient training and optimization relying heavily on large-scale, high-quality datasets from multiple sources [1-3]. However, the acquisition and sharing of medical data face significant obstacles, including privacy concerns, data security risks, and regulatory constraints [4].

In the digital medicine era, the secure exchange and control of sensitive health information have become central concerns in modern health care systems [5,6]. Meanwhile, strict legal and regulatory requirements—such as HIPAA (Health Insurance Portability and Accountability Act) and GDPR (General Data Protection Regulation)—must be met when handling these personal data [7]. Additionally, the competition between different medical centers and hospitals has led to data often remaining siloed [8]. These challenges not only impede the effective integration of multisource health care data but also hinder the translation of ML models from theoretical research to clinical practice.

While conventional centralized data storage architectures can partially facilitate model development, their dependence on centralized infrastructures has become increasingly problematic, rendering them vulnerable to single-point failures and malicious cyberattacks [9,10]. Consequently, establishing secure frameworks for cross-institutional data sharing and intelligent processing—while rigorously protecting data security and patient confidentiality—has emerged as a pivotal challenge impeding progress in medical AI. The fundamental tension between aggregating health care data for scientific progress and preserving individual privacy and data security has spurred the development of novel computational approaches. Federated learning (FL), as an emerging ML paradigm, addresses part of this problem by allowing institutions to collaboratively train models without exchanging raw data [11,12]. However, in practice, the deployment of FL in health care faces practical obstacles: the integrity and authenticity of model updates may be subject to security threats, including malicious attacks, client-side data tampering, and model forgery—all of which can reduce the accuracy of the global model. Furthermore, at present, there is a lack of reliable incentive structures for continuous participation, with limited auditability. The heterogeneity between institutions and edge devices undermines the integration and generalization of the model. Blockchain technology, with its decentralized architecture, immutable ledger system, and transparent traceability, has become a potential solution to address these limitations of logical reasoning [2,13,14]. When the two are integrated, blockchain can provide a verifiable source for model contributions, automated and transparent incentive mechanisms [15,16], tamper-proof logs for auditing, and a governance layer that supports cross-institutional workflows [17]. Therefore, the combined paradigm of blockchain-based federated learning (BCFL) is expected to become a practical approach to coordinating data privacy, trust, and collaborative intelligence in medicine.

Although research on FL and blockchain has accelerated in recent years, existing reviews exhibit clear gaps in focus and depth, limiting their value for clinical researchers and multidisciplinary audiences. The main shortcomings can be summarized as follows. First, most reviews examine either FL or blockchain technology in isolation, without providing a systematic analysis of how these two approaches can be integrated to address concrete challenges in health care. Second, prior reviews tend to emphasize algorithmic and technical details, while offering limited discussion of how BCFL can be adapted to real-world medical scenarios—such as cross-hospital electronic health record (EHR) integration, collaborative training of medical imaging models, Internet of Medical Things (IoMT) device coordination, and epidemic surveillance. Finally, few reviews adequately address the challenges, regulatory considerations, and future development trends of BCFL within the constraints of modern health care governance frameworks.

To address these gaps, this review makes three key contributions. First, it provides a medical demand–oriented, systematic classification of BCFL frameworks and outlines their typical workflows. Second, it maps different BCFL architectures to representative health care application scenarios, clarifying their practical relevance. Third, it analyzes the technical, regulatory, and implementation challenges that currently hinder BCFL adoption and identifies promising directions for future research, providing evidence-based insights for clinical translation and decision-making in the health care and biomedical research communities.

### Objective

Although previous studies have analyzed BCFL frameworks from the perspectives of technology and commercial applications, the review of their practical application scenarios in health care remains limited. Especially in the era of AI, with the explosive growth of medical data, the application of large models in medical practice has encountered some development obstacles, bringing some new ideas for the application prospects of BCFL in health care. This study investigates the synergistic integration of FL and blockchain technology, elucidating their combined architectural framework and operational mechanisms. We demonstrate how this technological convergence enables secure, privacy-preserving health care data sharing and collaborative model development across distributed health care institutions. Although blockchain-enhanced FL has recently emerged as a promising approach in medical research, the field remains nascent, constrained by technical limitations, unresolved privacy issues, and implementation barriers. Furthermore, we present a systematic review of current advancements in blockchain-assisted FL for medical applications and propose actionable research directions to overcome existing challenges.

Key contributions of this work include:

1. This study systematically reviews the theoretical foundations of FL and blockchain technology and elaborates on their potential and advantages in the medical field.
2. A comprehensive taxonomy of existing integration frameworks, accompanied by a mechanistic analysis of bidirectional benefits: how blockchain enhances FL security and trustworthiness, and how FL expands blockchain’s utility in distributed computing scenarios.
3. To summarize recent advancements and practical applications of BCFL across key health care domains, including cross-institutional medical data sharing, IoMT, public health surveillance, and telemedicine.
4. The current technological limitations and application challenges are examined, and key future research directions are proposed to address these gaps and advance real-world implementation.

By synthesizing current research and offering a structured analytical framework, we aim to provide a comprehensive reference for medical personnel, researchers, and health care policymakers. Ultimately, it fosters the development of trustworthy, privacy-preserving, and collaborative AI systems that can support precision medicine and smart health care in a decentralized digital era.

## Methods

### Overview

This review systematically summarizes recent advances in BCFL within the medical field, following the PRISMA (Preferred Reporting Items for Systematic Reviews and Meta-Analyses) checklist (Checklist 1). To ensure methodological rigor and reproducibility, we adopted a transparent multistep process including comprehensive literature search, independent screening, quality assessment, and evidence synthesis.

### Search Strategy

The literature data were primarily retrieved from several prominent academic databases, including PubMed, IEEE Xplore, Web of Science, and Google Scholar. To ensure the timeliness of the research, the review focuses on literature published between January 2018 and February 2025, while also incorporating some early seminal studies to trace the theoretical development and technological evolution of BCFL in the medical field.

After that, the search strategy uses Boolean logic operators to formulate a comprehensive formula, for example, (“blockchain”) AND (“federated learning”) AND (“medical” OR “healthcare”). To enhance retrieval efficiency and encompass interdisciplinary intersections, the search terms are further expanded, such as “(blockchain-enabled federated learning),” “(distributed machine learning),” “(decentralization),” “(Internet of Medical Things (IoMT),” “(telemedicine),” “(EMR),” and “(epidemics)” are incorporated to ensure literature comprehensiveness. The exact search string is as follows: (“blockchain” OR “distributed ledger technology”) AND (“federated learning” OR “collaborative learning” OR “distributed machine learning”) AND (“healthcare” OR “medical” OR “clinical” OR “EMR” OR “(epidemics)” OR “IoMT” OR “telemedicine”). Gray literature (conference proceedings and preprints from arXiv) was screened manually and included only if it contained original data or detailed technical methodology relevant to BCFL.

### Inclusion and Exclusion Criteria

To ensure relevance and academic rigor, this review establishes strict inclusion and exclusion criteria. The inclusion criteria are as follows: (1) the studies must involve blockchain and FL technologies and explore their medical applications; (2) the literature published in peer-reviewed journals and reviews indexed in the Science Citation Index or Social Sciences Citation Index or in top-tier international conferences (eg, IEEE and Association for Computing Machinery) and seminal papers with >30 citations were included regardless of publication venue; and (3) reported theoretical frameworks, system architectures, empirical evaluations, or case studies. The exclusion criteria then include (1) studies focusing solely on blockchain or FL without medical applications, (2) editorial and opinion articles that lack technical details or empirical validation, and (3) duplicate reports or low-quality publications from non–peer-reviewed sources.

### Literature Screening and Processing

The above search strategy initially retrieved 2547 documents. After automatic deduplication by EndNote, 1327 were retained. Subsequently, two independent reviewers (XW and XC) manually screened all the literature in two stages based on the inclusion and exclusion criteria. First, based on the title and abstract, high-quality reviews, papers from top journals and conferences, and literature that clearly explained the application of BCFL in the health care field were selected, totaling 319 articles. In the second stage, further review was conducted. Through full-text reading, studies lacking empirical verification, technical details, or experimental data were eliminated, and ultimately 111 high-quality documents were retained. In addition, to ensure the comprehensiveness of the literature, this review also referred to the latest review papers and included the core research results cited therein to avoid missing key progress. Detailed literature retrieval strategies can be found in Multimedia Appendix 1. After the screening process, the reviewers evaluated the quality of the literature. Since the corpus of this review mainly comes from the cross-disciplinary research of medicine, engineering, and computer science, the GRADE (Grading of Recommendations Assessment, Development, and Evaluation) framework commonly used in clinical evidence-based studies was not applicable. We used the Systematic Literature Review quality checklist based on the Kitchenham principle [18] to score each of the 12 indicators of the included studies, including reproducibility, method transparency, evaluation design, data description, experimental validity, attack/privacy discussion, and reproducibility, item by item (1/0.5/0). Two independent reviewers (XW and XC) assessed each document. When there were significant differences in the scores (the total score difference of a single document was greater than 2 or there were differences in key items), a third senior reviewer (YX) arbitrated. Ultimately, the studies were classified into three quality grades based on the total score: high (≥9), medium (5-8), and low (<5). When writing summaries and conclusions, priority was given to citing high-quality research. Multimedia Appendix 2 [1-130] provides complete assessment criteria, scoring rules, and statistics on peer-to-peer agreements.

## Results

### Study Findings

**Figure 1.**
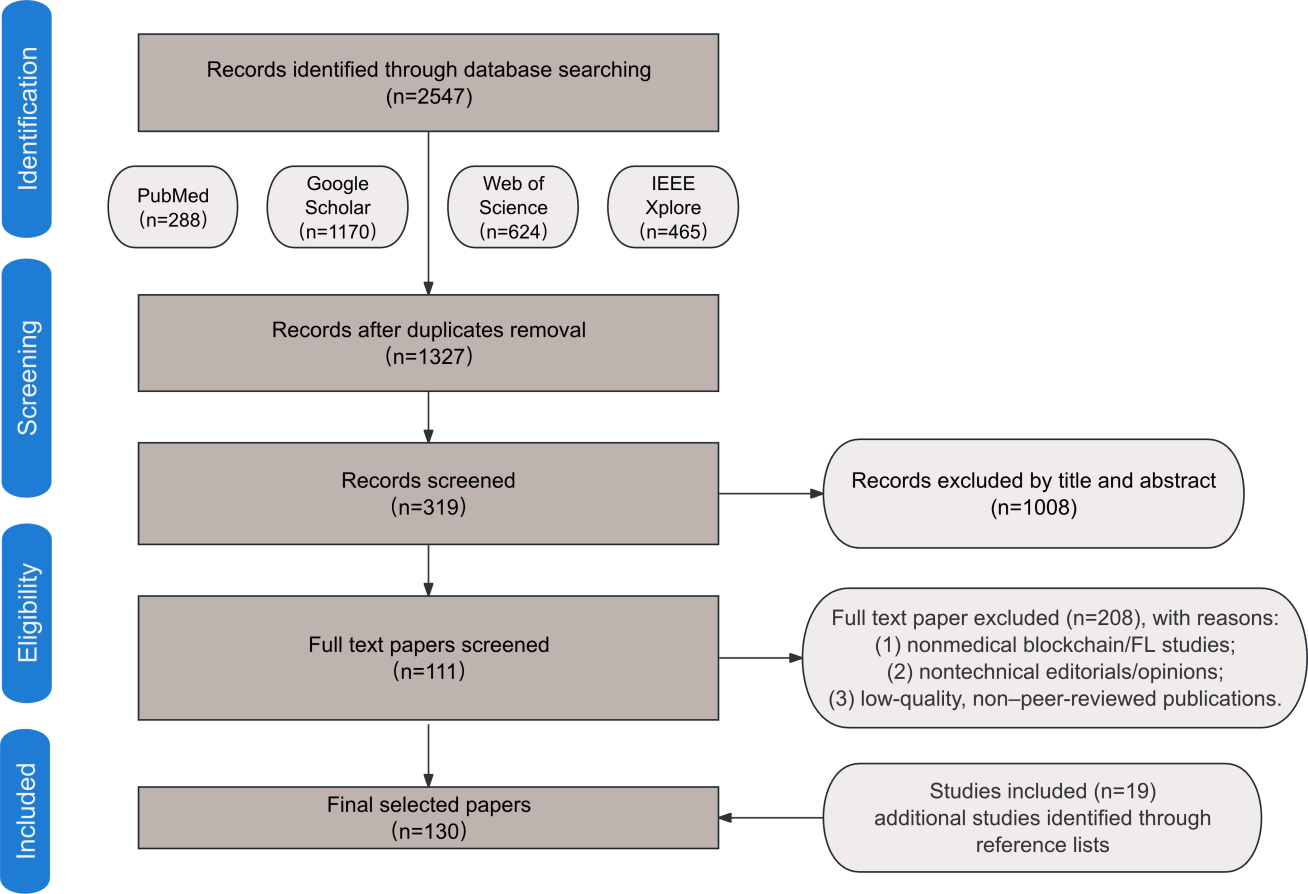
PRISMA (Preferred Reporting Items for Systematic Reviews and Meta-Analyses) flow diagram of the systematic review phases. FL: federated learning.

Finally, this review is founded on over 100 strictly selected papers, encompassing theoretical research, technical architecture, application scenarios, challenges, and future trends. These studies provide a robust academic foundation for an in-depth exploration of BCFL applications in medicine. A PRISMA flow diagram illustrates the systematic selection process (Figure 1).

### Current Status of Artificial Intelligence in Medicine

In the context of the rapid advancements in AI, the performance of ML models heavily depends on access to large volumes of high-quality data. However, the health care sector has long struggled with data sharing due to concerns over privacy, security, and regulatory compliance. The prevalence of “data silos” in health care impedes the advancement of AI and hinders the translation of research findings into practical clinical applications.

Traditional centralized ML approaches require aggregating raw data from multiple sources to a central server for training. While this approach allows for extensive data usage, it still presents significant challenges in practical implementation. First, data stored and transmitted in a centralized system is susceptible to network attacks, which can lead to sensitive information theft or tampering, posing a serious privacy risk. Second, centralized storage faces significant compliance challenges, especially under stringent privacy regulations such as GDPR [7] and HIPAA, which further restrict the cross-organizational exchange of health care data. Moreover, health care organizations, research institutions, and enterprises are often reluctant to share critical data due to competitive concerns and resource protection, thus further exacerbating data silos and hindering cross-organizational collaboration in building high-quality ML models.

### Federated Learning in Health Care

In this context, the limitations of traditional algorithms are becoming increasingly apparent, driving researchers to explore innovative solutions. In 2016, Google introduced the concept of federated learning [19], a distributed and collaborative ML paradigm. At its core, FL enables multiple data holders to collaboratively develop a global model by training locally and exchanging model parameters without sharing raw data. This paradigm is well-suited for scenarios with strict privacy requirements and decentralized data that cannot be centrally stored, such as health care, finance, and smart cities [20-23]. This approach not only effectively enhances data privacy protection but also overcomes the limitation of data silos, ushering in a new era of privacy-preserving collaborative learning.

**Figure 2.**
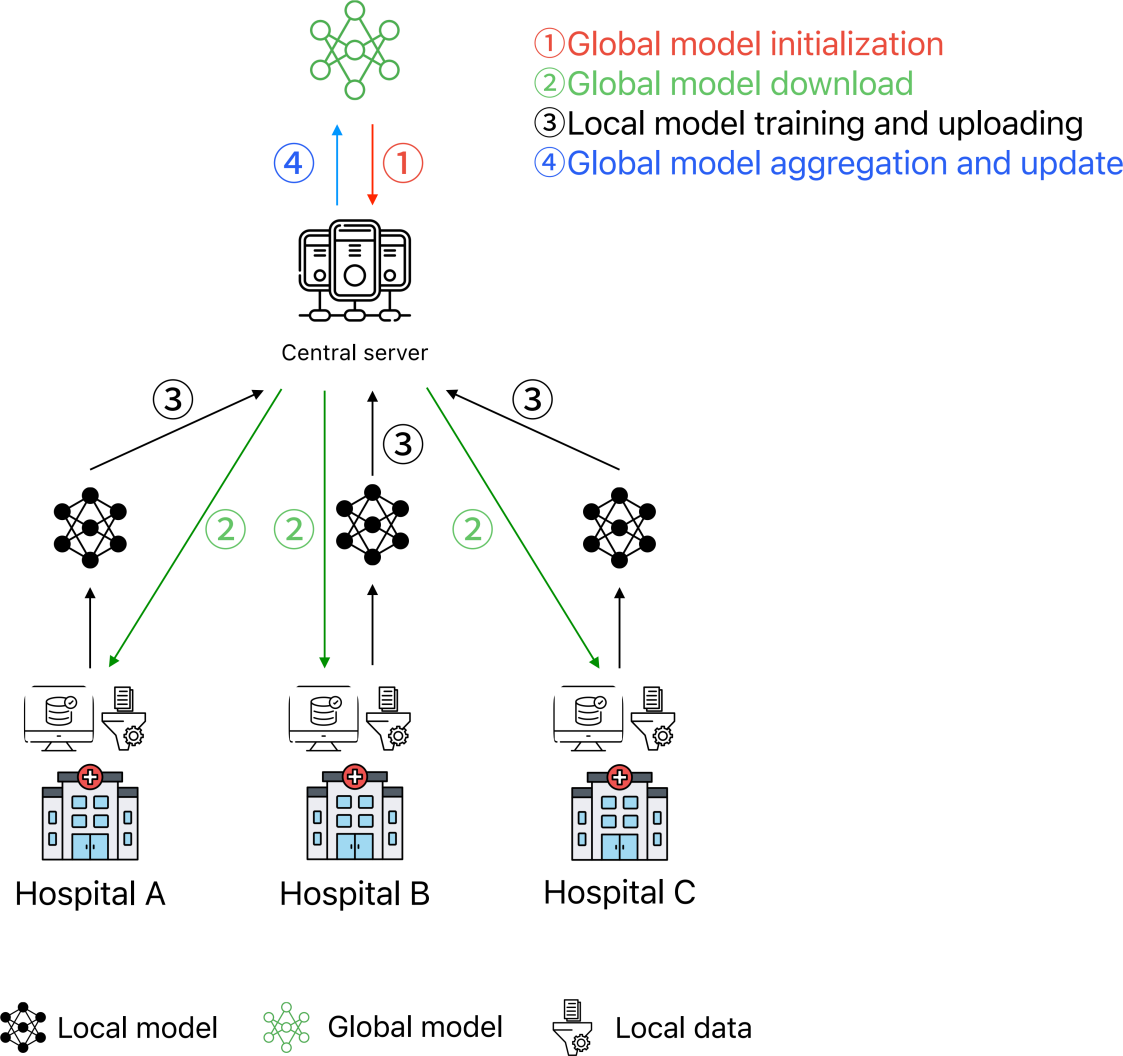
Federated learning architecture and workflow in the medical field.

FL operates on the principles of distributed model training and global parameter aggregation. One of its core algorithms is the Federated Averaging (FedAvg) algorithm. As illustrated in Figure 2, the fundamental process can be summarized as follows [24,25]. The first step is that a central coordination server initializes the global model and distributes it to all participating clients (eg, hospitals or mobile devices). Then each client trains the model locally using its private dataset and transmits the updated model parameters (or gradients) back to the central server in an encrypted form. Following this, the central server collects and aggregates the model parameters uploaded by all clients and updates the global model based on a predefined aggregation algorithm (eg, FedAvg). The updated model is then redistributed to the clients for the next training round. This process is iteratively performed over multiple communication rounds until the global model converges or predefined performance metrics are met.

Based on the above operating principle of FL, its unique technical characteristics give it different advantages in the health care field: if it can use large-scale, diverse, and geographically distributed datasets without compromising patient privacy, this decentralized approach not only mitigates the impact of data silos, which are common in hospitals and research institutions, but also captures a broader range of features by leveraging heterogeneous real datasets, ultimately achieving an AI model with higher efficiency, robustness, and accuracy. Therefore, since its proposal, FL has shown great application potential in the field of health care [26]. For instance, FL has been applied in drug research, allowing pharmaceutical companies to leverage shared algorithmic models to accelerate drug discovery while avoiding direct data sharing [27]. Additionally, FL-based collaborative protocols, involving multiple hospitals and cloud servers, have been developed for EHR analytics [28]. Moreover, FL has been investigated for predicting hospitalizations in cardiac patients [29]. FL has also been widely studied in medical imaging applications, such as prostate cancer detection, brain tumor segmentation [30], and MRI analysis for Alzheimer and Parkinson diseases. Moreover, FL-based approaches have been proposed for detecting coronavirus infections [31,32].

Despite the promise of FL in medical applications, its practical application still faces the following challenges: a central concern is its dependence on a centralized server for model coordination and parameter aggregation. However, the central server can be a source of a single point of failure and remains susceptible to man-in-the-middle attacks [33,34]. Moreover, as an increasing number of local devices simultaneously transmit model parameters to the central server, it places significant pressure on server bandwidth and scalability, thereby elevating the risk of network congestion [8,35,36]. Besides, although FL avoids direct sharing of raw data, its training process may still inadvertently expose sensitive information through model parameters. For instance, via gradient inversion attacks [37], adversaries can reconstruct sensitive training data from shared model parameters. The risk is further heightened by the presence of malicious participants, who may compromise the integrity of FL by uploading falsified or adversarial local training updates, such as poisoning attacks [38]. These attacks degrade the reliability of the global model, reducing its accuracy and overall utility. Additionally, the lack of a robust incentive mechanism poses a practical barrier to adoption. FL implicitly assumes that all participants willingly contribute data and computational resources without direct compensation. However, this assumption is difficult to uphold in real-world scenarios. The absence of a fair and transparent incentive mechanism may diminish participants’ motivation to contribute high-quality updates, ultimately degrading system performance [39-41]. Moreover, some participants may receive rewards without actively contributing data, leading to unfair financial compensation.

When FL is applied to the medical domain, heterogeneity across systems, data, and distributions presents a fundamental challenge to model reliability and practical deployment. System heterogeneity arises from disparities in computing power, memory, network stability, and energy availability among participating devices [42]. This is particularly critical in resource-constrained environments, where limited hardware capabilities and intermittent connectivity can hinder local training and delay model updates. Data heterogeneity further complicates collaboration, as medical institutions often store data in different formats and standards, leading to integration difficulties [43,44]. Variations in data quality—such as incomplete records or inconsistent annotations—and significant differences in dataset sizes across institutions can distort the learning process and reduce model performance. Most critically, distribution heterogeneity, or the non-independent and identically distributed (non-IID) data problem, undermines generalizability. Institutions serve distinct patient populations, resulting in divergent feature and label distributions; for example, a model trained predominantly on “healthy” samples may struggle with accurate predictions when exposed to datasets with a higher prevalence of “diseased” samples, reducing its generalizability.

These advantages and limitations together underscore the urgent need for complementary technologies to enhance trust, security, and coordination in FL workflows. Blockchain, with its decentralized, tamper-resistant, and auditable infrastructure, offers a promising solution to many of these pain points. In the following section, we explore how blockchain can be integrated with FL to build more robust, transparent, and privacy-preserving frameworks for medical applications.

### Blockchain in Health Care

Introduced in 2008 as the foundational technology behind the Bitcoin system, it was originally designed to solve trust-related issues in digital currency transactions [45]. Essentially, it functions as a distributed digital ledger, where transaction records are maintained and shared across all participants through a peer-to-peer network. Unlike traditional centralized systems, blockchain eliminates reliance on a trusted third party, ensuring decentralized data storage [46].

**Figure 3.**
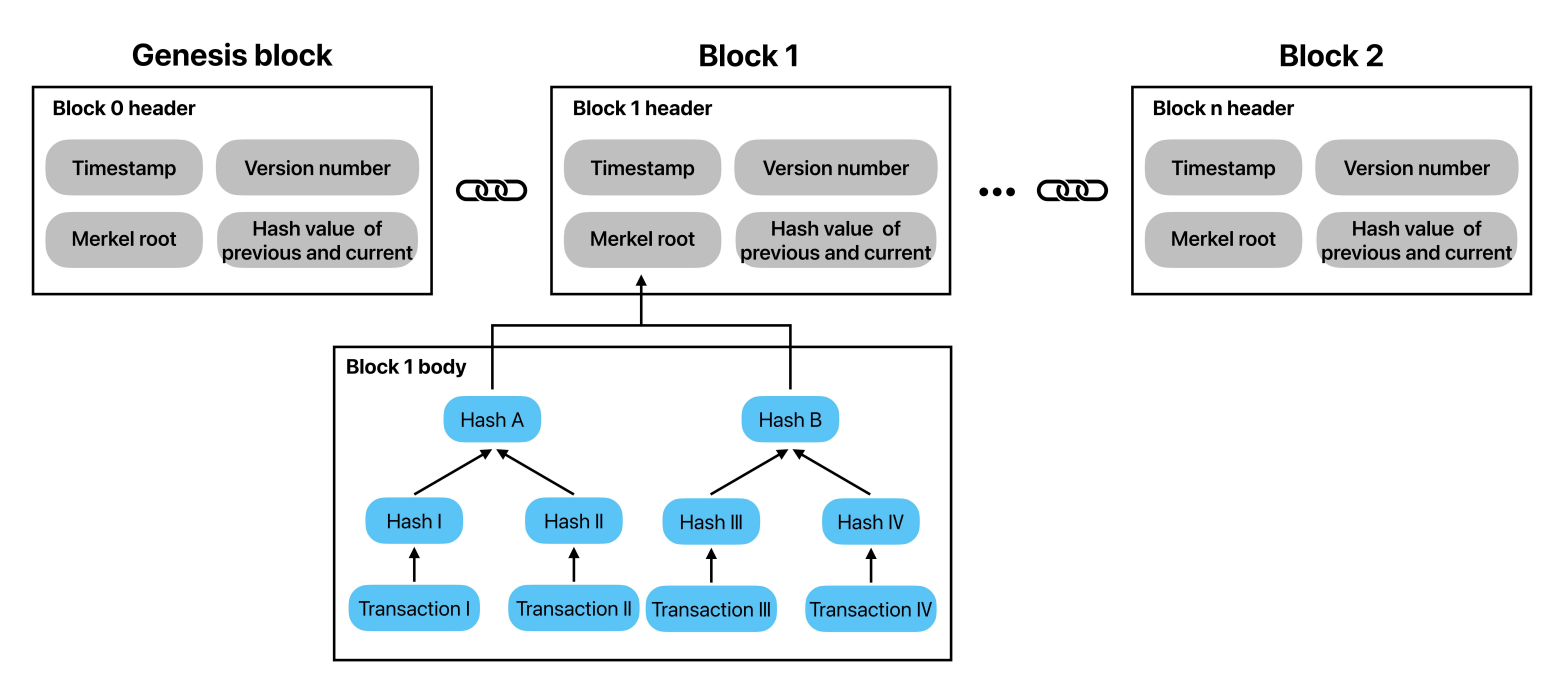
A schematic diagram of the blockchain structure.

The implementation of blockchain technology relies on multiple technical layers and core components. To fully understand the principles of blockchain, it is essential to analyze its specific components. The fundamental unit of blockchain is the “block,” comprising two primary components: the block header and the block body, which is shown in Figure 3. The block header contains metadata, including the hash value of the preceding block (which links the current block to its predecessor), timestamps, random numbers, and block version numbers. This metadata establishes the linkage between blocks. The block body holds smart contracts and actual data, including transaction records. These blocks are cryptographically linked in chronological order, forming an immutable chain structure. Each block is closely linked to its predecessor via a hash value, ensuring that any tampering with a single block will alter the hash values of all subsequent blocks. This change is detectable and will be rejected by the entire network, thereby preserving data authenticity and integrity.

**Figure 4.**
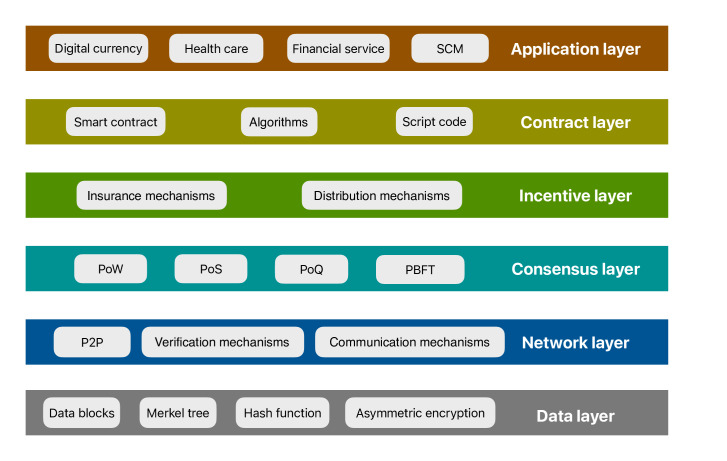
The system architecture of blockchain. P2P: Peer to Peer; PBFT: Practical Byzantine Fault Tolerance; PoQ: Proof of Quality; PoS: Proof of Stake; PoW: Proof of Work; SCM: Supply Chain Management.

Furthermore, to fully understand the potential of blockchain in such applications, it is essential to examine its underlying architecture and systematically analyze its functional layers (shown in Figure 4).

Fundamentally, blockchain systems are structured into 6 tightly interwoven layers, each addressing specific functional needs. At the core lies the data layer, which serves as the foundation for storing and organizing transaction records. The data layer organizes and secures transaction records through cryptographic techniques. This layer not only guarantees data integrity and immutability but also significantly reduces storage overhead, which is critical for handling the growing volume of medical data [47]. Above this, the network layer enables efficient and reliable information exchange across decentralized nodes via peer-to-peer communication protocols [47]. In the context of high-stakes environments like health care, securing consensus across distributed and often mutually untrusting participants is the responsibility of the consensus layer. By establishing agreement on the validity and chronological order of transactions, consensus mechanisms prevent malicious interference and ensure the reliability of shared records [48,49]. The widely adopted algorithms include Proof of Work (PoW), Proof of Stake (PoS), and Practical Byzantine Fault Tolerance (PBFT) [16,50,51]. To sustain long-term network participation, the incentive layer introduces economic mechanisms that reward nodes for validating transactions and generating new blocks. This is especially important in medical applications, where incentivized behavior facilitates the uploading of more high-quality medical data, while the authenticity and availability of data directly affects patient outcomes. Building upon these foundations, the contract layer operationalizes complex protocols through smart contracts, which autonomously execute predefined rules and transactions without the need for third-party intermediaries [52]. In the health care sector, this translates into streamlined workflows for processes such as insurance claims, consent management, and secure data access, offering enhanced transparency, reduced administrative overhead, and minimized human error. Finally, the application layer serves as the interface between users and the blockchain, bridging technical functionality with real-world applications. Across industries, this layer has fueled innovations in financial services, supply chain management, and beyond [53,54]. In health care, it enables secure sharing of EHRs, protecting patient privacy, and supporting collaborative research efforts across institutions [55]. By facilitating trusted interactions in environments where data sensitivity and security are paramount, blockchain’s multilayered architecture offers an indispensable foundation for integrating advanced paradigms such as FL, thereby unlocking new possibilities for privacy-preserving, distributed medical intelligence.

Through the analysis of the structure and underlying architecture of blockchain, we can better understand that blockchain has unique characteristics that distinguish it from traditional systems, including decentralization, immutability, transparency, traceability, security, anonymity, and high availability [8,13].

These characteristics are particularly valuable in the field of health care. For instance, decentralization eliminates the inherent single point of failure in centralized medical record systems, thereby enhancing the system’s resilience and reducing its vulnerability to cyber attacks. This feature is particularly suitable for scenarios with high trust requirements, such as medical record management and supply chain supervision. Immutability and traceability not only ensure that clinical data, diagnostic results, and patient consent records cannot be altered or deleted, guaranteeing the auditing of medical data, but also are crucial for tracking the source of drugs and ensuring the integrity of the supply chain, thereby reducing the risk of counterfeit drugs. Furthermore, blockchain offers a transparent and privacy-protecting environment that enables authorized clinicians, researchers, and insurance companies to verify data sources without exposing sensitive patient information, thereby enhancing accountability in medical research and drug development. The security of blockchain stems from smart contracts, consensus mechanisms, etc, maintaining transaction integrity and preventing malicious nodes from arbitrarily altering the block addition process [56,57].

As illustrated in Multimedia Appendix 3, a comparison of the technical characteristics of blockchain and FL is presented. Simultaneously, given these characteristics, blockchain facilitates trust establishment among untrusted participants in wireless networks [58,59]. Consequently, blockchain demonstrates great potential across various domains, including cryptocurrencies, health care, and the Internet of Things (IoT) [45,60,61]. In health care, blockchain technology enables the storage and verification of IoT data within patients’ electronic medical records (EMRs), clinical trials, and sensors, granting patients control over their own medical data [62-64]. During AI-driven sample learning, medical data from various institutions—including x-rays, CT scans, MRI reports, and pathological examinations—are securely stored on the blockchain. Predefined entity and event annotation platforms facilitate data labeling within each institution, followed by model training on in-hospital servers [65,66]. Kordestain et al [67] proposed HapiChain, a telemedicine platform built on a patient-centered blockchain infrastructure, ensuring the security of remote consultations between patients and doctors. In addition, decentralized blockchain solutions, such as Drug-ledger and Med-ledger, have been proposed to enhance traceability and security in the pharmaceutical supply chain [68,69].

Given blockchain’s significant advantages in decentralization, privacy protection, data immutability, incentives, and automation, its attributes fit well with FL’s requirements for secure data sharing and distributed modeling. Blockchain can serve as a secure and reliable collaborative infrastructure for FL, addressing challenges such as trust deficiency, data integrity, and transparency [4,22,70]. Conversely, the decentralized data processing mechanism of FL can compensate for blockchain’s limitations in scalability and computational efficiency. Therefore, integrating blockchain with FL has the potential not only to address their respective challenges but also to unlock new application scenarios and possibilities. The following section examines the necessity and feasibility of integrating blockchain with FL.

### Integration of Blockchain and Federated Learning in Health Care

#### Overview

BCFL has emerged as a promising solution in health care, fostering a balance between privacy protection and data collaboration while unlocking new opportunities for data-driven health care innovation [71,72]. The following section analyzes the complementary strengths and performance of integrating blockchain with FL, illustrating how blockchain mitigates the limitations of FL (as illustrated in Multimedia Appendix 3) and how FL benefits blockchain.

#### Blockchain Empowers Federated Learning in Health Care

##### Decentralization Mitigates Single Points of Failure and Scalability Bottlenecks

The traditional architecture of FL primarily depends on a central server to manage and coordinate participants. This architecture is susceptible to single points of failure and can also result in bandwidth and computational resource bottlenecks as the number of clients increases [70,73,74]. In contrast, blockchain’s decentralized architecture eliminates reliance on a central server by leveraging a distributed network, allowing automated data collaboration across multiple nodes and effectively mitigating the risk of single points of failure [13]. In this context, temporary aggregators are selected based on blockchain’s consensus mechanisms (eg, PoW or PoS) [8,36]. Moreover, blockchain’s Byzantine fault tolerance, which enables the dynamic management of unreliable nodes through consensus mechanisms, further enhances the stability of FL in large-scale distributed environments [75].

##### Incentives to Enhance Participant Motivation

Traditional FL systems often lack effective incentives, especially in environments where resources are unevenly distributed. High-performing participants may struggle to sustain long-term contributions due to the absence of direct benefits [76,77]. Blockchain’s built-in incentive mechanisms address this challenge by rewarding data contributors, validators, and maintainers through tokens or other financial instruments, introducing an economic driver for FL [78]. This incentive model encourages the contribution of high-quality data while discouraging unreliable participation, ultimately improving the overall performance and stability of FL models. For instance, Weng et al [79] proposed an incentive mechanism designed to promote collaboration in training deep learning models. The mechanism introduces two key concepts: compatibility and activity. Compatibility ensures that each participant receives optimized rewards based on their contribution, while activity incentivizes participants to update the local model and aggregate the global model actively. Upon each global model update, rewards are distributed to local devices and miners based on individual contributions. Similarly, Kang et al [77] introduced a reputation-based incentive model to measure client trustworthiness. By leveraging blockchain’s immutability, the system ensures distributed reputation management and evaluates participants based on model quality and computational contributions.

##### Privacy Protection and Attack Resistance Enhancement

Although FL protects raw data privacy, it remains vulnerable to adversarial threats such as poisoning attacks and Byzantine attacks, which can mislead model training and hinder convergence [36,80]. Blockchain strengthens FL security through its immutability and tamper-proof nature. Its authentication mechanisms detect and exclude malicious nodes, ensuring that only authorized participants can access FL data, thereby enhancing privacy protection. Moreover, blockchain’s cryptographic techniques and anonymity mechanisms reduce the risks of background knowledge attacks and collusion attacks [78]. Additionally, its consensus mechanism ensures data consistency across all nodes while using sophisticated algorithms to prevent malicious nodes from compromising the network [81,82]. For example, in medical data collaboration, blockchain records the training processes and contributions of each participating hospital, ensuring both data integrity and privacy while mitigating risks of data contamination and adversarial attacks. Shayan et al [83] proposed a multi-Krum consensus mechanism to counter poisoning attacks by electing a validation peer committee that filters out malicious model updates. Similarly, Chen et al [84] used a blockchain-based validation voting mechanism, where nodes vote on model update validity and remove malicious devices based on consensus results.

##### Transparency and Auditability

Blockchain’s transparency and auditability effectively address the challenges of trust deficits and compliance difficulties in FL. By leveraging blockchain’s transparent data-sharing mechanisms, FL participants can verify the source and integrity of health care data and model updates in real time, ensuring fairness and reliability in contributions [72]. Additionally, blockchain’s immutable records establish an accountability framework for FL [33,85]. These records facilitate anomaly detection and responsibility attribution throughout the model training process, strengthening compliance and governance mechanisms. This transparent and auditable nature not only fosters trust in collaborative learning but also provides a technological foundation for regulatory compliance.

##### Automated Management With Smart Contracts

Smart contracts enable automated execution of key processes in FL, including model update sharing, model update validation, and global model aggregation. By enforcing predefined rules, smart contracts eliminate human intervention, ensuring an unbiased and tamper-proof process. Moreover, they can dynamically allocate resources and rewards through conditional triggering mechanisms—such as when a model reaches an expected accuracy or when a node successfully completes a specific task [86,87]. This automation enhances the autonomy and reliability of FL while ensuring transparency and fairness through open code logic. By reducing administrative overhead and mitigating trust concerns, smart contracts introduce a novel and efficient approach for managing decentralized collaborative learning [33].

### How Federated Learning Can Benefit Blockchain

#### Enhancing Blockchain Consensus Efficiency

The blockchain consensus mechanism, while ensuring network security and data consistency, is often associated with substantial computational costs and energy consumption, a challenge that is particularly serious in the PoW mechanism [8,88]. PoW relies on miners solving complex hashing operations to compete for block generation, necessitating the continuous operation of high-performance computing hardware, which in turn results in substantial global energy consumption. Studies indicate that the annual energy consumption of the Bitcoin network is comparable to that of a small- to medium-sized country. This highly inefficient competition leads to an enormous waste of computational resources—only the first miner to discover a valid hash can package the transaction and claim the reward, rendering all other computational efforts futile. Furthermore, the excessive energy consumption of PoW constrains blockchain’s sustainable adoption in critical domains such as health care data management and edge computing, necessitating the development of more energy-efficient consensus optimization strategies. In this context, FL offers a novel approach to optimizing blockchain consensus mechanisms. Integrating the blockchain consensus process with FL allows miners to contribute to model training while competing for block validation, effectively repurposing computational resources that would otherwise be wasted. This approach not only reduces energy consumption but also enhances computational resource efficiency, rendering the consensus process more practically valuable [89].

#### Facilitating Cross-Chain Data Collaboration

As blockchain applications continue to expand, the demand for data exchange across different blockchains has grown, making cross-chain technology a crucial solution to addressing “data silos” across various domains. FL and its variants, such as federated transfer learning, can establish a unified model collaboration framework across different blockchain networks, enabling privacy-preserving data sharing and joint modeling. By maintaining a shared ML model, disparate blockchains can collaborate while preserving autonomy and privacy, thereby facilitating cross-chain applications in finance, health care, and other sectors [90].

#### Enhancing Blockchain Scalability

Blockchain faces storage and computational bottlenecks when handling large-scale data. FL, by adopting a local training model that eliminates the need to upload raw data to the blockchain, significantly reduces on-chain storage demands. Additionally, FL alleviates blockchain’s computational burden by distributing processing tasks among participating nodes, thereby providing a scalable foundation for large-scale collaboration.

### Architectural Frameworks for Integrating Blockchain and Federated Learning

A BCFL typically adopts one of three architectural paradigms: fully coupled, flexibly coupled, and loosely coupled architectures [41,85,91]. These architectures differ in terms of the degree of coupling between blockchain nodes and FL clients, each offering unique characteristics in function allocation, resource usage, and system structure.

#### Fully Coupled BCFL

The fully coupled architecture represents a highly integrated design, wherein FL clients simultaneously function as blockchain nodes, assuming dual roles. Consequently, each node is responsible for local model training, update validation, global model aggregation, and new block generation. These tasks are executed on a single node, fostering a fully decentralized collaborative model [36,74,79,92].

Global model aggregation can be carried out either by selected nodes or collaboratively by all nodes, depending on the network’s design strategy. Moreover, the blockchain’s distributed ledger not only records local model updates but also stores global models and other relevant information generated during training, ensuring data integrity and traceability.

#### Flexibly Coupled BCFL

The flexibly coupled architecture achieves higher design flexibility by separating FL clients from blockchain nodes. In this architecture, FL clients primarily handle local data collection and model training, whereas blockchain nodes are responsible for validating model updates, storing the global model, and maintaining the ledger [2,40,93]. The blockchain can aggregate global models via selected nodes, which typically possess superior computing resources and reliability, thereby reducing resource consumption and enhancing system efficiency. Alternatively, aggregation can be performed collaboratively by all nodes, achieving full decentralization and mitigating the risk of a single point of failure.

This architecture significantly lowers the resource requirements for FL clients, allowing them to function in different network environments while preserving blockchain’s inherent advantages in data security and transparency. Due to its high adaptability, this architecture has become a preferred choice for large-scale distributed systems, such as health care data sharing and cross-organizational collaboration.

#### Loosely Coupled BCFL

The loosely coupled architecture further weakens the coupling between blockchain nodes and FL clients by optimizing functional allocation. FL clients primarily perform local model training and upload updates to the blockchain for validation, whereas the blockchain handles authentication, model update validation, and participant reputation management.

In this architecture, the blockchain does not store model updates but instead records only reputation-related data. A reputation mechanism is implemented as a key criterion for assessing participant reliability, thereby incentivizing them to contribute high-quality data and updates [77,94,95]. This design enhances system scalability by alleviating storage pressure on the ledger while ensuring the trustworthiness of participant behavior.

### Workflow in BCFL

#### Overview

In BCFL systems, the flexibly coupled architecture has emerged as the predominant choice for real-world applications due to its optimal balance of efficiency and adaptability. By separating FL clients from blockchain nodes, this architecture allows them to operate on different networks and devices, thereby reducing system communication overhead and latency. Additionally, it alleviates the computational and storage burden on client devices while preserving key advantages such as data privacy protection and blockchain-based verification, ultimately achieving an optimal balance between efficiency and privacy. Leveraging these advantages, the flexibly coupled architecture has demonstrated significant potential in practical applications, including medical data sharing and cross-organizational collaboration.

**Figure 5.**
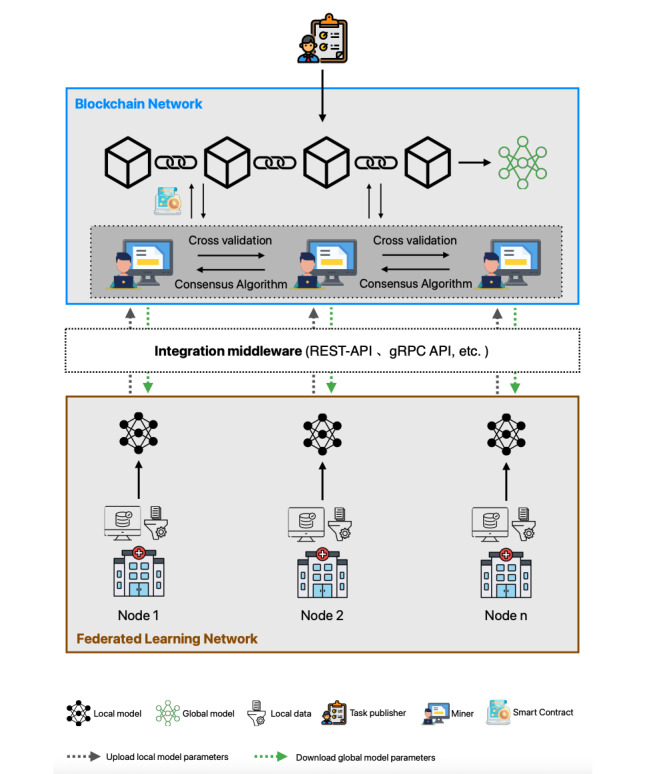
Flexibly coupled blockchain-based federated learning architecture and workflow. gRPC API: Google Remote Procedure Calls – Application Programming Interface; REST-API: Representational State Transfer – Application Programming Interface.

As illustrated in Figure 5, the following section focuses on the specific workflows of mainstream BCFL frameworks, analyzing their distinct advantages in practical applications.

#### Task Release

Task initiators release FL tasks and requirements on the blockchain, specifying details such as data volume and type, hardware specifications, and the number of training rounds. Leveraging blockchain’s transparency and decentralization, this process ensures fair and open task distribution while fostering participant trust.

#### Local Model Training and Update Transmission

Each FL client downloads the initial global model from the blockchain, after which it preprocesses local data, extracts features, and uses this data for model training, subsequently generating local model updates. These updates are then transmitted to the blockchain network in encrypted form.

It is important to note that, in the flexibly coupled architecture, FL clients and blockchain nodes operate within different networks and systems, each with clearly defined responsibilities. Therefore, this architecture heavily relies on integrated middleware, which serves as a communication bridge and coordinator between the two components. In a research study, Lamken et al [96] used REST-API (Representational State Transfer – Application Programming Interface) for communication with the Hyperledger Fabric blockchain, facilitating the recording and incentivization of gradient uploads. Additionally, the Remote Procedure Calls (RPC) protocol developed by Google, known as the gRPC API, facilitates data exchange between FL clients and the Ethereum blockchain network [33,96].

#### Blockchain Node Verification Update

Blockchain nodes (ie, miners) verify the uploaded model updates using a predefined validation mechanism. Concurrently, miners exchange their validated local model updates with each other. A consensus algorithm guarantees that only validated updates contribute to the global model aggregation.

#### Global Model Aggregation

Subsequently, the blockchain selects an interim leader among its nodes through a consensus mechanism. The selected node(s) then collect verified model updates and aggregate them to construct the global model [36]. The flexibly coupled architecture enables this process to be executed by selected nodes or collectively by all nodes, thereby opening up the possibility of optimizing efficiency across various scenarios.

#### New Block Generation and Model Storage

Validated model updates and global models are packaged by selected blockchain nodes to generate new blocks. Upon adding the block header information, the legitimacy of the block is verified through a consensus mechanism among the nodes.

#### Distributed Ledger Update

The newly generated blocks are broadcast across the entire network, and all blockchain nodes update their local ledgers after verification. This process ensures the transparency and traceability of the global model and its associated information throughout the network.

#### Reward Distribution and Incentives

The system allocates rewards, such as cryptocurrency or reputation scores, based on client performance. This incentive mechanism not only motivates participants to contribute high-quality updates but also deters malicious behavior, thereby enhancing the accuracy and reliability of the model.

#### Global Model Download

After the training is completed, all participating clients can download a newly generated block containing the updated global model parameters from the blockchain. Clients can then independently decide whether to participate in the next training round based on their specific needs. This mechanism enhances both system flexibility and participant autonomy.

As illustrated in Table 1, a comparison of BCFL integration architectures is presented. In future practical applications, the selection of a specific architecture must be carefully evaluated based on scenario requirements, resource constraints, and design objectives to achieve optimal collaboration and technical performance.

**Table 1.** Comparison of blockchain-based federated learning integration architectures.

Architecture type	Characteristics	Advantages	Disadvantages	Applicable scenarios
Fully coupled BCFL^a^	High integration: FL^b^ clients and blockchain nodes are fully merged Fully decentralized: All nodes work together through a consensus mechanism	High transparency: All transactions and model updates are recorded on the blockchain Strong security: Resistant to single-point failures and man-in-the-middle attacks Strict control: Highly controlled over data and models	High resource demand: Requires significant computational and storage resources High network complexity: All nodes participate in the consensus mechanism, and the network complexity is high Intensive coordination: Frequent internode communication is required	Large-scale distributed environments: Suitable for large medical institutions and research centers Strict control and security requirements: Scenarios involving the sharing and analysis of sensitive medical data [97,98]
Flexibly coupled BCFL	Functional separation: FL clients operate independently from blockchain nodes Computational offloading: Model aggregation occurs at selected nodes	Enhanced efficiency: Optimized allocation of computing and storage resources Greater flexibility: Can be adapted to different application scenarios Improved scalability: Supports large-scale data sharing and collaborative learning	Complex coordination: The responsibilities of clients and nodes are separated, and complex coordination and management mechanisms are required Centralization risks: Use of a centralized aggregator may introduce a single point of failure Node selection challenges: Issues such as node selection criteria and fairness are involved	Dynamic collaboration settings: Suitable for cross-institutional medical data sharing, IoMT^c^ device management [40,99]
Loosely coupled BCFL	Minimal integration: FL clients and blockchain nodes operate independently Lightweight blockchain: Primarily used for identity authentication and reputation management	Reduced overhead: Reduce the operating cost of blockchain and improve system performance Enhanced privacy: Reduce on-chain storage pressure and improve scalability Optimized incentives: Reputation-based mechanisms encourage high-quality contributions	Lower decentralization: May still rely on trusted central nodes for model aggregation Data integrity risks: Blockchain does not store model updates	Resource-constrained environments: Suitable for wearable medical devices and real-time health monitoring Small-scale institutions: Ideal for personal mobile health applications and smaller clinics [100]

a BCFL: blockchain-based federated learning.

b FL: federated learning.

c IoMT: Internet of Medical Things.

### BCFL in Medicine

#### Overview

**Figure 6.**
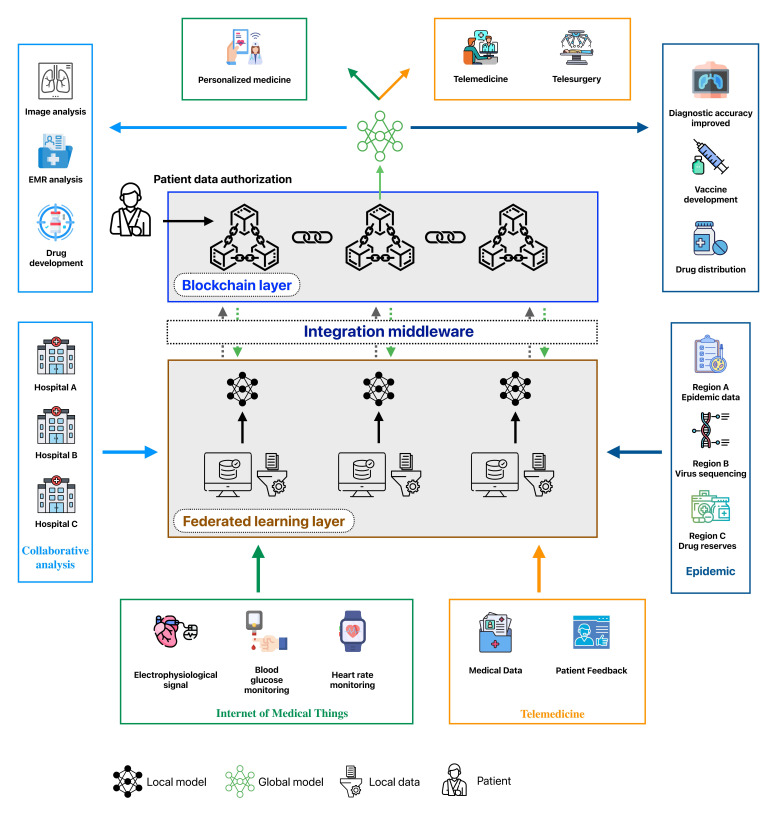
Blockchain-based federated learning framework for different domains in health care. EMR: electronic medical record.

As the demand for data-driven technologies in health care continues to grow, the BCFL framework presents significant potential due to its advantages in privacy preservation, data security, and collaborative efficiency. BCFL facilitates cross-organizational data sharing and collaborative analytics, optimizing personalized health care solutions while driving advancements in areas such as telemedicine, IoMT, and public health monitoring, which are shown in Figure 6. In the following section, we will discuss the various applications of BCFL in the medical field and analyze its key role and potential value in addressing real-world challenges.

#### Cross-Institutional Medical Data Sharing and Collaborative Analysis

In modern health care, data serves as a crucial resource for driving innovation and enhancing treatment efficacy. However, data sharing among health care institutions is hindered by concerns over privacy, data security, and regulatory compliance. The integration of blockchain and FL offers an innovative solution for cross-institutional health care data sharing and collaborative analysis. While numerous studies have demonstrated the feasibility of BCFL in various medical domains, the strength of evidence supporting these applications varies considerably, and critical challenges remain.

Several studies have investigated BCFL in the context of chronic disease management, particularly diabetes prediction. Hasan et al [101] developed a blockchain-FL framework that reported a 15% improvement in predictive performance across multiple metrics. Although these results are encouraging, the framework relied primarily on public diabetes datasets with limited diversity, raising questions about its generalizability to heterogeneous real-world populations. Similarly, Moulahi et al [102] evaluated a BCFL model on the Pima Indians Diabetes dataset, achieving a multilayer perceptron accuracy rate of 97.11% and an average FL accuracy rate of 93.95% while protecting privacy. Yet, the reliance on small, well-characterized datasets constrains the robustness of the findings. Taken together, these studies suggest that BCFL holds promise for chronic disease prediction, but the supporting evidence remains preliminary, and large-scale multi-institutional validation is still lacking.

In the realm of the IoMT, Ramani et al [103] introduced the ODMSM-FL (Optimized Data Management and Secured Federated Learning) approach to address secure data storage and exchange using EHR datasets from HealthData.gov. This research report presents a set of numerical results on key performance indicators: transaction throughput (102.75 Kbps), data retrieval delay (64.02 ms), security (88.97%), and accuracy (86.32%). The research results highlight the great potential of ODMSM-FL in effectively addressing the urgent data management and security issues in IoMT. However, the system was evaluated under controlled experimental conditions rather than real-world clinical settings, limiting its immediate applicability. By contrast, research in medical imaging tasks, such as brain tumor segmentation, has placed greater emphasis on model accuracy and privacy preservation. For example, Kumar et al [104] proposed a permissioned blockchain-based federated framework with quality-aware model aggregation, achieving improved segmentation metrics on the BraTS 2020 dataset. Specifically, compared with the baseline method, our approach increased the Dice similarity coefficient of enhanced tumors by 1.99% and reduced the Hausdorff distance of the overall tumor by 19.08%. Although the study demonstrated methodological innovation, it still relied on benchmark imaging datasets rather than prospective clinical data, which restricts the strength of evidence regarding its clinical translatability.

Other investigations have targeted specific diagnostic applications. Heidari et al [105] designed the FBCLC-Rad (Federated Learning–Enabled Blockchain CapsNets Lung Cancer Radiologist) framework for lung cancer detection, achieving near-perfect accuracy on nodule identification tasks. This technology achieved an accuracy rate of 99.69% with the lowest classification error. While technically impressive, results derived from controlled datasets may not fully reflect the complexity of real-world diagnostic workflows. Liang et al [106] extended BCFL applications to clinical trials, where blockchain ensured data authenticity and traceability, and FL supported participant screening across organizations. This study represents an important step toward integrating BCFL into the clinical research pipeline, but it remains largely conceptual, with limited empirical validation in actual trial environments.

A growing body of work has also highlighted the integration of BCFL with EMRs to facilitate precision medicine [99,107-110]. This approach has demonstrated significant effectiveness in enhancing diagnostic accuracy, optimizing treatment planning, identifying patient subgroups for clinical trials, and accelerating the development of novel therapeutics. Within the paradigm of precision medicine, such a framework facilitates a transition from the traditional “one-size-fits-all” treatment model to a more personalized and adaptive intervention strategy. However, despite their conceptual appeal, most studies are limited to prototype frameworks or simulations and have not yet undergone prospective evaluation in clinical practice. As such, the current evidence supporting BCFL in EMR-based precision medicine remains promising but immature.

Overall, existing literature demonstrates the conceptual feasibility and technical potential of BCFL for cross-institutional health care data sharing and analysis. Nevertheless, the evidence base is uneven: studies using small public datasets provide only preliminary support, while those addressing more complex tasks such as imaging or clinical trials often lack real-world validation.

#### Internet of Medical Things

The IoMT is an advanced technological ecosystem that integrates internet technology with medical devices, enabling real-time data collection, exchange, and analysis to enhance clinical decision-making, disease prevention, and patient care. With the rapid advancement of the IoMT, the traditional hospital-centric model has evolved into a patient-centered health care system driven by comprehensive clinical analysis. The widespread adoption of IoMT enables individuals to conveniently monitor their health at home, thereby streamlining diagnosis and treatment while allowing patients to enjoy more efficient, personalized health care. However, despite its growing adoption, IoMT in health care is also facing significant challenges. One of the primary concerns is data privacy and security. IoMT devices collect and transmit vast amounts of sensitive data, including patient identities, insurance details, and payment information. Once these data are accessed by malicious individuals, it could lead to serious consequences. Moreover, the absence of standardized security protocols among IoMT devices exacerbates the risks of data leakage and device manipulation. Consequently, device manufacturers and health care institutions face immense pressure to ensure data privacy and regulatory compliance. Additionally, IoMT devices frequently encounter challenges such as high computational complexity, elevated costs, and communication delays due to resource limitations.

To address these limitations, several studies have explored the integration of blockchain and FL in IoMT. For instance, Rahman et al [111] proposed a lightweight hybrid FL framework that leverages blockchain to secure health data provenance and uses smart contracts to coordinate model training and trust management. While this framework demonstrates theoretical scalability and robust traceability, its evaluation was primarily conducted in simulated settings, raising concerns about its applicability in heterogeneous and large-scale real-world health care environments.

Muazu et al [112] combined BCFL with edge computing to improve resource allocation, reduce computational costs, and enhance IoMT data security. By offloading intensive computations to edge nodes, the study reported reductions in latency and energy consumption. Meanwhile, the performance of the proposed model offers a higher precision of 83% and an accuracy rate of 78%. However, the framework largely relies on linear regression as the global learning model, which—although interpretable and useful for basic clinical predictions—may not adequately capture the complexity of real-world multimodal medical data. Compared with Rahman et al [111], this work provides stronger performance evidence in terms of latency and efficiency but weaker generalizability for complex clinical prediction tasks.

Dhasaratha et al [113] extended BCFL by incorporating reinforcement learning and distributed computing to improve risk factor monitoring and COVID-19 patient prediction. The dynamic optimization enabled by reinforcement learning is a notable strength, allowing adaptive performance improvements in evolving environments. Compared with the previous two documents, this approach offers methodological novelty but lacks equally rigorous performance benchmarking across standard IoMT metrics.

To tackle fraud detection and scheduling issues, Lakhan et al [114] introduced the FL-BETS (Federated Learning–Based Blockchain-Enabled Task Scheduling) framework, which integrates BCFL with dynamic heuristics for task scheduling across fog and cloud nodes. The experimental results show that the framework, from the initial 60:80 fraud delay ratio to a 10:10 ratio, demonstrates better performance in energy-delay trade-offs and antifraud behavior. Yet, the framework emphasizes technical efficiency rather than clinical utility, and its reliance on hard and soft scheduling constraints may limit adaptability in unpredictable medical environments. Compared with Dhasaratha et al [113], which focuses on patient-specific outcomes, this work provides stronger technical validation but weaker clinical alignment.

In the context of wearable IoMT devices, Baucas et al [115] designed a system that integrates FL with a private blockchain in a fog computing architecture to enhance privacy and adaptability. Their framework demonstrated efficiency in resource-constrained environments and produced accurate predictive models while safeguarding patient privacy. Unlike the literature above, this study directly validated its framework on wearable health care devices, thereby providing more immediate clinical relevance. However, the scalability of the approach for larger IoMT networks remains uncertain.

Overall, these studies collectively highlight the potential of BCFL in overcoming IoMT’s inherent privacy, security, and performance limitations. Yet, their evidence strength varies significantly: some emphasize theoretical frameworks validated in simulations [111], while others demonstrate more robust experimental performance [112,114] or closer alignment with clinical practice [115].

#### Public Health Surveillance and Epidemiological Forecasting

The global outbreak of COVID-19 highlighted the limitations of existing surveillance infrastructures, particularly the inability to provide accurate, real-time epidemic monitoring. Traditional methods often struggle with the rapid spread and variability of epidemics, while stringent privacy requirements hinder effective collaboration across institutions. For instance, during global outbreaks such as COVID-19, the inability of national and regional health care organizations to efficiently integrate data has impeded comprehensive analyses of epidemic progression [116]. This phenomenon of data silos delays the formulation of precise response strategies and undermines the efficiency of vaccine distribution and health care resource allocation. Therefore, achieving efficient and secure data integration while preserving privacy has emerged as a critical challenge in public health. The BCFL framework not only integrates anonymized health data from diverse regions but also facilitates the efficient construction of predictive models for epidemic spread. This framework enables multiple health care organizations and research institutions to collaborate securely without compromising patient privacy, thereby providing robust data support for early epidemic detection, transmission trend analysis, and the formulation of intervention strategies. However, the strength of evidence supporting BCFL frameworks varies considerably across different studies, depending on data scale, validation methods, and implementation feasibility.

FedMedChain [117] represents an early attempt to address these challenges. By combining blockchain with FL and leveraging the IoMT, it enhances the trustworthiness of public health communication and mitigates risks associated with centralized data transmission. Its contribution lies in demonstrating that blockchain can ensure data transparency and tamper resistance while maintaining privacy. Nevertheless, FedMedChain was mainly verified through small-scale simulation experiments rather than real-world deployments, which limited the strength of the evidence and its direct clinical applicability.

In contrast, Kumar et al [118] explored a BCFL framework for processing heterogeneous CT images using capsule networks. This method achieves a high detection accuracy on the CC-19 dataset, and its research results include 98.68% specificity and 98% sensitivity. While the study demonstrates the feasibility of applying BCFL to medical imaging and highlights the benefits of privacy-preserving collaboration, the restricted dataset size and limited institutional diversity weaken its external validity. Compared with FedMedChain, this workplaces greater emphasis on model performance but provides weaker evidence regarding scalability and generalizability.

Durga and Poovammal [119] extended this direction by proposing the FLED-Block (Federated Learning–Ensembled Deep Learning Blockchain Model) framework, which integrates blockchain with FL for COVID-19 prediction using multisource heterogeneous CT datasets. This framework improves the classification accuracy by using capsule networks for feature extraction and extreme learning machines for efficient classification. The research results include an accuracy of 98.2%, a precision of 97.3%, and a recall rate of 96.5%. Importantly, it integrates blockchain to share model weights without the need to exchange raw data, thereby resolving privacy issues. Compared with Kumar’s study [118], FLED-Block was supported by evidence from more hospitals, providing stronger validation. However, the latency of blockchain is regarded as a limitation, raising questions about its applicability in real-time clinical diagnosis. Therefore, although this framework demonstrates outstanding technical performance, its transformation in emergency health care settings remains uncertain.

Abdel-Basset et al [120] proposed the blockchain-based federated learning for pandemic diagnosis (BFLPD) framework, which stands out for its focus on system security and robustness in the context of smart cities. Unlike FedMedChain and FLED-Block, BFLPD combines more encryption technologies, including secure aggregation, homomorphic encryption (Cheon-Kim-Kim-Song scheme), and consensus mechanisms (PBFT), to mitigate malicious attacks and improve reliability. The classification accuracy of BFLPD reaches 95.14%, exceeding the benchmark set by the most advanced distributed models. In addition, this framework also demonstrated significant precision and recall rates (95.26% and 95.77%, respectively) and a relatively high *F*_1_-score (95.52%). Meanwhile, the authors incorporated heat map visualization, further enhancing its clinical application value. This framework provides stronger evidence than earlier works, as it addresses adversarial threats that are often overlooked in BCFL studies. Nevertheless, its reliance on complex cryptographic and consensus algorithms introduces implementation challenges, such as high computational overhead, which could hinder real-world deployment.

#### Telemedicine and Telesurgery

In recent years, telemedicine has experienced rapid advancements, especially in response to the global COVID-19 pandemic, which has significantly increased its role in modern health care systems. Telemedicine leverages modern information and communication technologies to facilitate medical information exchange across geographic boundaries, encompassing various applications such as remote diagnosis, remote consultation, remote treatment, and continuous health monitoring [5]. By providing on-demand, personalized health care services, telemedicine optimizes medical resource allocation, effectively addressing the challenge of unequal distribution of traditional health care resources and ensuring medical support for patients in remote or underserved areas. Despite its potential, telemedicine faces several critical challenges in practical implementation:

- Data Security and Privacy Risks: Most telemedicine systems rely on centralized cloud servers to store patient health data, making them vulnerable to single points of failure.
- Lack of Data Access Control Mechanisms: Many existing telemedicine platforms do not offer a robust data access control framework, meaning that once patient data are uploaded to the cloud, patients often lose ownership and control over their own health records.
- High Infrastructure and Computational Costs: Telemedicine demands substantial computational resources, high-speed communication networks, and specialized medical equipment, particularly for real-time diagnosis and treatment.

Building upon these advantages, recent studies have proposed generalized frameworks that integrate blockchain and FL to support secure and scalable telemedicine systems. For example, Hiwale et al [5] highlighted the importance of incorporating privacy-preserving technologies into BCFL, laying the groundwork for reliable, privacy-compliant telemedicine applications. Within such frameworks, blockchain’s distributed ledger technology enables decentralized data storage, reducing the risks of single points of failure and data breaches, while ensuring transparency and traceability in data access. Simultaneously, FL enhances data privacy by enabling local model training, thereby minimizing the exposure of sensitive health information. Although valuable as a theoretical framework, this study provides limited experimental validation and thus represents weak evidence for clinical applicability. Gupta et al [121] enhanced trust between patients and providers by designing a smart contract system based on public blockchains. The framework allows patients to retain ownership and fine-grained control of health data, addressing a central shortcoming of conventional telemedicine platforms. Compared to Hiwale et al [5] conceptual work, Gupta et al [121] system offers a more concrete mechanism for authorization and data sharing. Nonetheless, its validation remains restricted to simulation environments, with no real-world deployment or clinical evaluation. As such, while it provides moderate evidence of feasibility, its generalizability remains uncertain.

With the rapid advancement of technology and the increasing improvement of medical demands, traditional telemedicine models are evolving beyond routine diagnosis and treatment. Among these advancements, telesurgery—a critical extension of telemedicine—is emerging as a transformative innovation. However, this technology imposes stringent requirements on real-time data synchronization, precise coordination of surgical equipment, and robust data security, introducing new challenges to the reliability of underlying technological infrastructures. For instance, Chaudjary et al [122] proposed a secure telesurgery system that integrates blockchain and FL with 6G communication networks and the Interplanetary File System protocol. This study demonstrated notable improvements in latency reduction, storage efficiency, and transmission reliability compared with traditional telesurgery systems. Unlike earlier works by Hiwale et al [5] and Gupta et al [121], Chaudhary et al [122] provided more systematic experimental results, suggesting stronger evidence of technical feasibility. However, these results were still derived from controlled simulations rather than real-world surgical environments, and issues such as blockchain latency and computational overhead remain unresolved. Therefore, while the study represents the strongest evidence among current works, its translation into clinical practice requires further validation.

In the past few years, BCFL’s research in the health care field has shown significant growth. Multiple studies have confirmed the improvement of model performance in public datasets or experimental environments, such as enhancing the accuracy of disease prediction, strengthening image diagnostic capabilities, or improving edge device management.

However, when examined from the perspective of evidence, most of these achievements are still at the stage of simulation experiments, prototype systems, or preclinical validation. Research is usually based on controlled datasets or static data scenarios, and there is a significant gap between the model performance and the actual clinical diagnosis and treatment process. Importantly, current literature pays more attention to technical indicators (such as accuracy, Dice, and delay) rather than specific medical endpoints, such as changes in misdiagnosis rates, shortened treatment duration, or improved patient prognosis. Therefore, a direct chain of evidence has not yet been formed between the technical performance of BCFL and its actual medical value.

Furthermore, the BCFL architecture is not inherently compatible with the real deployment environment. The medical system features a complex governance structure, compliance requirements, and heterogeneous infrastructure. However, existing research often assumes node autonomy, network stability, or institutional equivalence, while neglecting key issues such as data authorization, responsibility division, and system compatibility. Although blockchain consensus, smart contracts, and high-intensity encryption enhance security, they also bring about latency, energy consumption, and maintenance costs, which conflict with the real-time and reliability requirements of clinical practice. These mismatches have led to many frameworks performing well in experimental settings but being difficult to migrate to real medical scenarios.

Given the above limitations, relying solely on technical performance indicators cannot accurately reflect the maturity of BCFL in medical scenarios. To more systematically assess the application level of existing research, we adopted an evidence stratification strategy to categorize the existing literature from multiple dimensions such as architectural innovation, deployment environment, verification depth, clinical relevance, and potential risks. This stratification aims to reveal the gap between “technical performance” and “medical practice value,” identify the most critical bottlenecks in the process from conceptual framework to clinical validation, and provide directional references for future research design. Table 2 summarizes the evidence stratification of typical BCFL studies in the health care field.

**Table 2.** Evidence stratification of blockchain-based federated learning studies in health care.

Reference	Application domain	BCFL^a^ architecture/contribution	Deployment environment	Validation depth	Clinical relevance/risk	Evidence level/maturity
[101]	Cross-Institutional Medical Data Sharing (Chronic Disease)	Proposed a decentralized and privacy-preserving collaboration framework that integrates blockchain and FL^b^, enhancing the predictive performance of diabetes models while ensuring data security and reducing communication overhead	Evaluation on public dataset (unspecified diabetes data)	Retrospective data validation	Population heterogeneity is not covered; it is difficult to extrapolate to real clinical patients	Level 3: Preclinical
[102]	Cross-Institutional Medical Data Sharing (Chronic Diseases)	Developed a blockchain-integrated FL mechanism to enhance IoMT^c^ data privacy and improve diabetes prediction accuracy, achieving 97.11% accuracy with a multilayer perceptron model	Evaluation on public dataset (Pima Indians Diabetes)	Retrospective data validation	Relying on a small and well-defined dataset limits the robustness of the findings	Level 3: Preclinical
[103]	Cross-Institutional Medical Data Sharing (Data Management)	Introduced the ODMSM-FL^d^ framework, which optimizes storage, management, and privacy protection for IoMT data, enhancing data security and system efficiency	Evaluation on public EHR^e^ dataset (HealthData.gov)	Controlled experimental conditions	Data latency, human-machine device heterogeneity; The real IoMT network is uncontrollable	Level 2: Prototype validation
[104]	Cross-Institutional Medical Data Sharing (Medical Imaging)	Designed a blockchain-powered FL framework for brain tumor segmentation using 3D U-Net, achieving significant improvements in Dice similarity coefficient and Hausdorff distance	Evaluation on public benchmark dataset (BraTS 2020)	Retrospective data validation	Relying on benchmark datasets rather than prospective clinical data limits the clinical translational application	Level 2: Prototype validation
[105]	Cross-Institutional Medical Data Sharing (Medical Imaging)	Proposed the FBCLC-Rad^f^ framework, integrating CapsNets, blockchain, and FL to enhance lung cancer nodule detection accuracy in CT scans, reaching 99.69% accuracy	Evaluation on public and local dataset (Cancer Imaging Archive [CIA], Kaggle Data Science Bowl [KDSB], LUNA 16, and local datasets)	Retrospective data validation	The process of not covering the real image; lack of doctor decision-making and workflow verification	Level 2: Prototype validation
[106]	Cross-Institutional Medical Data Sharing (Drug Discovery)	Designed Rahasak-ML, a decentralized blockchain-FL platform enabling multi-institutional collaboration with enhanced transparency and security in drug discovery	Theoretical analysis; limited empirical validation	Conceptual framework	Empirical verification in actual test environments is limited	Level 1: Conceptual
[107]	Cross-Institutional Medical Data Sharing (EMR^g^)	Integrated FL and blockchain for cloud-based medical record recommendation systems, leveraging Hyperledger Fabric, IPFS^h^, LightGBM, and N-Gram models for collaborative learning	Evaluation on public EHR dataset (not specified)	Simulated	Limited to prototype frameworks or simulations, not prospectively evaluated in clinical practice	Level 2: Prototype validation
[108]	Cross-Institutional Medical Data Sharing (EMR)	Proposed a blockchain-FL framework for EHR privacy protection, achieving 92.5% global model accuracy and 88.33% local model accuracy using a deep neural network	Evaluation on public EHR dataset (Chronic Kidney Disease [CKD] dataset [UCI Machine Learning Repository])	Retrospective data validation	Limited to prototype frameworks or simulations, not prospectively evaluated in clinical practice	Level 3: Preclinical
[109]	Cross-Institutional Medical Data Sharing (EMR)	Used lightweight encryption and FL to secure EHR data in an Ethereum test environment, reducing reliance on trusted third parties	Evaluation on public EHR dataset (Simulation in Ethereum test environment)	Simulated	Limited to prototype frameworks or simulations, not prospectively evaluated in clinical practice	Level 2: Prototype validation
[110]	Cross-Institutional Medical Data Sharing (EMR)	Combined CNN^i^ and blockchain-FL to enhance EHR data security and detect abnormal user behaviors automatically	Evaluation on public EHR dataset (Python-based simulation)	Simulated	Limited to prototype frameworks or simulations, not prospectively evaluated in clinical practice	Level 2: Prototype validation
[99]	Cross-Institutional Medical Data Sharing (EMR)	Explored blockchain-FL applications in precision medicine, emphasizing diagnostic accuracy, treatment optimization, clinical trial subpopulation identification, and drug development acceleration	Evaluation on public EHR dataset (not specified)	Simulated	Limited to prototype frameworks or simulations, not prospectively evaluated in clinical practice	Level 2: Prototype validation
[111]	IoMT (Data Security)	Proposed a lightweight hybrid FL framework with blockchain smart contracts for edge training plan management, trust evaluation, and authentication in IoMT networks	Evaluation on public COVID-19 dataset (not specified)	Simulated	The device has heavy computational burden, high system complexity, and difficult clinical translation	Level 1: Proof-of-concept
[112]	IoMT (Data Management)	Developed a blockchain-FL system leveraging edge computing and Paillier encryption to securely manage medical resource transactions in IoMT environments	Evaluation on public dataset (unspecified diabetes data)	Retrospective data validation	The device has heavy computational burden, high system complexity, and difficult clinical translation	Level 2: Prototype validation
[113]	IoMT (Data Security)	Introduced a distributed reinforcement learning method integrating blockchain and FL for improved data privacy and security in IoMT applications	Evaluation on public COVID-19 dataset (not specified)	Simulated	The device has heavy computational burden, high system complexity, and difficult clinical translation	Level 2: Prototype validation
[114]	IoMT (Data Security)	Proposed the FL-BETS^j^ framework, leveraging fog computing and blockchain to minimize energy consumption and latency while enhancing fraud detection in health care	Evaluation on Private dataset focusing on medical insurance fraud (Kaggle)	Simulated	The device has heavy computational burden, high system complexity, and difficult clinical translation	Level 1: Proof-of-concept
[115]	IoMT (Data Security)	Developed a fog computing IoT platform that integrates FL and private blockchain technology to enhance privacy protection in wearable IoMT devices	Evaluation on human activity recognition dataset (UCI^k^ Machine Learning Library)	Simulated	The device has heavy computational burden, high system complexity, and difficult clinical translation	Level 2: Prototype validation
[117]	Public Health (COVID-19 Imaging)	Proposed a blockchain-FL-based IoMT architecture for COVID-19 detection and epidemic management; the architecture enhances data privacy through FL and ensures data transparency and immutability via blockchain	Evaluation on public COVID-19 dataset (Centers for Disease Control [CDC] data)	Simulated	High real-time requirements in epidemic environment; blockchain delay is not resolved	Level 1: Proof-of-concept
[118]	Public Health (COVID-19 Imaging)	Developed a blockchain-based FL framework for COVID-19 detection, using Capsule Networks for image segmentation and classification to enhance data privacy and model accuracy	Evaluation on Private COVID-19 dataset (CC-19)	Retrospective data validation	The limited scale and types of data restrict the generalization ability of the model	Level 3: Preclinical
[119]	Public Health (COVID-19 Imaging)	Introduced FLED-Block^l^, a blockchain-based FL model integrating Capsule Networks for image feature extraction and extreme learning machines (ELM) for classification, achieving high accuracy with strong privacy protection	Evaluation on public COVID-19 dataset (CT data from multiple hospitals)	Retrospective data validation (multisource datasets)	The computational complexity and the feasibility of actual deployment require further research	Level 3: Preclinical
[120]	Public Health (Pandemic Diagnosis)	Designed BFLPD^m^, a blockchain-FL framework for epidemic diagnosis in smart cities, particularly for COVID-19; the framework ensures secure model aggregation and enhances global model integrity and efficiency	Evaluation on public ultrasound COVID-19 dataset (POCUS, ICLUS-DB, and COVIDx-US)	Retrospective data validation (multisource datasets)	The huge computational overhead and the high resistance to actual deployment	Level 3: Preclinical
[5]	Telemedicine (Telemedicine System)	Proposed a blockchain-FL application framework for telemedicine, analyzing how these technologies improve data accessibility, security, and privacy in remote health care	Based on theoretical simulations or examples	Conceptual framework	Mainly focuses on the theoretical framework, the lack of a real-world application cases	Level 1: Proof-of-concept
[121]	Telemedicine (Remote Surgery System)	An intelligent remote surgery framework named BITS^n^, which is based on blockchain and artificial intelligence, is proposed; this architecture integrates blockchain technologies (such as Ethereum and IPFS protocols), 6G communication networks, and federated learning (or AI^o^ algorithms), aiming to enhance the security, privacy, and real-time performance of remote surgery systems	Based on theoretical simulations or examples	Simulated	Limited to a simulated environment, no actual deployment or clinical evaluation has been carried out yet	Level 1: Proof-of-concept
[122]	Telemedicine (Remote Surgery System)	Developed a remote surgery system framework leveraging blockchain and FL to enhance data security, reliability, and real-time processing; the framework integrates 6G networks and IPFS for low-latency and high-reliability data transmission	Based on theoretical simulations or examples	Conceptual framework	There is insufficient discussion on the specific implementation details of BCFL in remote surgery and a lack of clinical deployment	Level 1: Proof-of-concept

a BCFL: blockchain-based federated learning.

b FL: federated learning.

c IoMT: Internet of Medical Things.

d ODMSM-FL: Optimized Data Management and Secured Federated Learning.

e EHR: electronic health record.

f FBCLC-Rad: Federated Learning–Enabled Blockchain CapsNets Lung Cancer Radiologist.

g EMR: electronic medical record.

h IPFS: Interplanetary File System.

i CNN: convolutional neural network.

j FL-BETS: Federated Learning–Based Blockchain-Enabled Task Scheduling.

k UCI: University of California, Irvine.

l FLED-Block: Federated Learning–Ensembled Deep Learning Blockchain Model.

m BFLPD: blockchain-based federated learning for pandemic diagnosis.

n BITS: Blockchain-Driven Intelligent Scheme for Telesurgery System.

o AI: artificial intelligence.

As shown in the table, most BCFL studies are still focused on the concept or prototype stage, lacking multicenter real data validation and evaluation corresponding to clinical endpoint indicators. To promote the clinical application of BCFL, improvements need to be made in three aspects: (1) conduct cross-institutional and prospective validations to evaluate the model’s performance in real patient populations and medical processes; (2) strike a balance among security, latency, and maintainability to avoid the unavailability caused by simply pursuing complex encryption or on-chain computing; and (3) achieve integration with medical information systems, data governance and regulatory frameworks, and deploy the system under the premise of clearly defining data responsibilities and authorities. Only when verified under the joint constraints of clinical workflow, patient heterogeneity, and compliance requirements can BCFL gradually evolve from a conceptual technology to a usable medical solution.

## Discussion

### Challenge

Although BCFL shows great potential in transforming health care data sharing, its deployment in real medical environments is still highly limited. The vision of change for BCFL must be balanced with technical limitations and the specific complexity of applications (particularly the interoperability gap, high implementation costs, and unresolved scalability bottlenecks), which prevent it from being transformed from a conceptual framework into regular clinical practice.

There are many application challenges in the field of medical data sharing, including the lack of system interoperability and the absence of standardized benchmark datasets specifically designed for medical applications. Currently, various health care information systems (eg, EMR systems) use diverse system architectures, data formats, and operational standards, lacking a unified interoperability framework. Moreover, unlike conventional FL research that often leverages open-access datasets such as CIFAR or MNIST, health care data are inherently sensitive, fragmented, and institution-specific, which makes reproducibility and cross-study comparability particularly difficult.

Scalability and communication efficiency also present critical obstacles. As FL tasks expand, the number of health care data sources and the complexity of training increase significantly. However, in practical deployments, the scalability and throughput limitations of blockchain are particularly pronounced. Even in permissioned frameworks such as Hyperledger Fabric, which offer improved throughput, empirical benchmarks still report end-to-end latencies of several seconds per block under moderate workloads. Insufficient mining resources slow down block generation and verification, hindering the efficient execution of large-scale tasks [123]. Moreover, the influx of numerous participants in distributed health care environments amplifies the load on the blockchain network, while the efficiency of existing consensus mechanisms is difficult to meet the demands of health care applications [124]. When applied to BCFL, these constraints imply that each iteration of local training, parameter aggregation, and block creation may introduce cumulative delays that significantly slow model convergence. Furthermore, as the number of blockchain nodes rises, communication costs increase exponentially. The network delays and communication efficiency degradation caused by high communication costs directly impact the training speed and overall model performance.

High implementation and maintenance costs represent another practical barrier. Establishing a BCFL infrastructure requires significant upfront investment in blockchain nodes, secure servers, storage, and high-speed networking. Additionally, energy consumption associated with blockchain consensus protocols, as well as the operational costs of managing frequent model updates across institutions, may exceed the financial capacity of many health care providers, especially in resource-limited settings. Without clear evidence of cost-benefit balance, hospitals and regulators may be reluctant to adopt BCFL at scale.

Additional difficulties arise from the integration of BCFL within IoMT environments. The heterogeneity of IoMT devices results in substantial disparities in storage capacity, computational power, energy consumption, and communication capabilities. For instance, advanced hospital equipment often features powerful processors, stable power supplies, and ample storage space, whereas wearable medical devices typically operate on low-power batteries, constrained network bandwidth, and limited computational resources. This disparity in device capabilities poses a significant challenge to the deployment of FL models. Moreover, energy constraints and unstable network connections make edge medical devices prone to data transmission failures or system disconnections, ultimately resulting in end-device desynchronization. This issue not only hampers the timeliness of data uploads and model updates but may also prevent the global model from converging efficiently.

Compounding these challenges is the heterogeneity of health care data within IoMT systems. Data generated by various devices exhibit significant diversity, often displaying uneven distributions and violating the non-IID assumption. For instance, hospital A may collect dynamic ECG signals, whereas hospital B primarily acquires static medical images. Such disparities in data distribution exacerbate the complexity of model training. Moreover, variations in medical coding standards across countries and regions (eg, ICD-10 [*International Statistical Classification of Diseases, Tenth Revision*] in the United Kingdom vs ICD-10-CM [*International Classification of Diseases, Tenth Revision, Clinical Modification*] in the United States) contribute to data standard inconsistencies. Consequently, such heterogeneities complicate global model training, analysis, and evaluation, ultimately impairing the model’s generalization across diverse clients [125].

Beyond the application-level challenges, BCFL also faces significant technical limitations that must be addressed to realize its full potential in medical settings. Although BCFL integrates the decentralized nature of blockchain with the “data availability without visibility” principle of FL to provide an initial level of privacy protection and enhance overall system security, it does not fully resolve privacy concerns. The sensitivity of patient data and the stringent privacy requirements in the medical field necessitate addressing a series of complex security challenges. The system remains vulnerable to various types of malicious attacks that pose significant risks to patient confidentiality and institutional trust. For instance, background knowledge attacks involve adversaries inferring sensitive information using previously known data and analyzing shared model parameters [3]. In conspiracy attacks, multiple nodes conspire to steal data features from other participants by exchanging local training information [126]. Inference attacks similarly analyze model parameter updates, and attackers can infer private details about patient data [1,43]. These threats highlight the critical need for advanced privacy-preserving mechanisms within BCFL to ensure the safety and integrity of medical data.

While existing privacy-preserving technologies provide preliminary protections, they are often inadequate when faced with the dual demands of strong privacy and high model utility. Homomorphic encryption allows computation directly on encrypted data, preventing plaintext exposure. However, its computational inefficiency makes it unsuitable for large-scale, complex operations. Differential privacy introduces noise to model parameters to obscure individual data contributions; however, this can significantly compromise model accuracy and system performance if not carefully balanced. Secure Multiparty Computation (SMPC) offers robust data confidentiality through distributed computation, yet its reliance on frequent interaction between parties contradicts the low-interaction protocols typically favored in BCFL for efficient aggregation. These limitations underscore the urgent need for lightweight, efficient, and scalable privacy-preserving solutions specifically tailored to the BCFL context.

Another critical technical issue is the optimization of incentive mechanisms. In traditional blockchain systems, fixed token-based reward structures fail to reflect the true value of each participant’s contribution [8]. This misalignment can result in low-quality nodes receiving undeserved rewards, while high-contribution nodes may become demotivated due to insufficient compensation. In BCFL, this issue is further complicated by the heterogeneity of participants, who differ in computational capabilities, data quality, and participation frequency. Resource-constrained nodes, in particular, may lack sufficient incentives to participate, ultimately affecting the quality and diversity of the global model. To address these disparities, incentive mechanisms in BCFL must go beyond simple token rewards and instead adopt dynamic, contribution-aware frameworks that account for the multidimensional nature of participant involvement. A well-designed incentive system can encourage broader and more sustained engagement, improve fairness, and enhance the overall efficiency and robustness of BCFL. Therefore, developing more sophisticated and adaptable incentive mechanisms is a pressing direction for future research.

While the immutability of blockchain and the tamper-resistant nature of smart contracts ensure data integrity and trustworthiness, these characteristics also introduce rigidity, posing challenges in dynamic health care environments [33]. Several scenarios highlight the importance for greater flexibility: when patient data are entered incorrectly or require modification, the immutable blockchain structure cannot accommodate efficiently; then patients may request the deletion or modification of their data to preserve privacy, particularly to comply with data protection regulations; moreover, in public health emergencies, ensuring timely access to accurate health care data is crucial for effective crisis management, necessitating mechanisms for controlled updates within blockchain systems. These scenarios underscore the need for greater flexibility within BCFL systems, where mechanisms must be designed to support controlled edits under predefined conditions—balancing the need for data integrity with the operational demands of evolving health care environments.

What’s more, another important consideration in the medical application of BCFL is model interpretability, which directly impacts the reliability of clinical decisions, patient trust, and regulatory compliance. In medical decision-making, the ability to interpret model outcomes is essential to ensuring safety, transparency, and credibility. However, the “black-box” nature of many complex deep learning models limits their interpretability, posing significant challenges for medical applications [106]. The necessity of interpretability can be emphasized from multiple perspectives: For health care professionals, AI model predictions must be interpretable to allow physicians to understand the underlying reasoning and effectively integrate them into diagnosis, treatment planning, and patient monitoring. Moreover, interpretability enables researchers and clinicians to identify and trace the sources of bias or errors in model predictions, facilitating performance optimization and improving diagnostic accuracy and reliability. Simultaneously, in terms of patients, the widespread adoption of AI in medicine inevitably raises concerns regarding privacy and ethics. Enhancing model interpretability can build patient trust in AI-assisted diagnosis and treatment by clarifying the model’s reliability and limitations, thereby mitigating concerns over “black-box” decision-making. Moreover, at the regulatory level, numerous countries and regions have mandated transparency and auditability in medical AI systems to ensure that decision-making processes align with ethical and legal standards. Furthermore, medical AI operates within a highly interdisciplinary environment, encompassing physicians, technology developers, data scientists, and other professionals. Interpretability serves as a crucial bridge for communication among experts from diverse disciplines, facilitating the effective implementation of medical AI technologies and ultimately enhancing the quality and accessibility of health care services.

Finally, summarize the limitations of the literature included in the review: first of all, the risk of prejudice is very common. Many available studies are conceptual frameworks, simulations, or small-scale case studies, rather than large-scale clinical implementations, without independent external validation. Common methodological flaws include the selective presentation of favorable performance metrics, limited or lack of adversarial and privacy attack tests, and the absence of long-term or actionable measurements (such as maintenance burden, interoperability failures, or ongoing participation rates). Unavailable code, undisclosed model/configuration details, and dependencies on nonshareable datasets often compromise reproducibility. In conclusion, these factors have created systemic uncertainties, posing the risk of overestimating feasibility and underestimating the actual deployment challenges. Second, the included studies demonstrated substantial inconsistencies in the key dimensions of system design and evaluation. Research in BCFL architecture (fully coupled, flexibly coupled, and loosely coupled), blockchain configuration (private and public), privacy countermeasures (secure aggregation, differential privacy, homomorphic encryption, and SMPC), and data sources (public benchmarks, single-center clinical records, and various IoMT streams) varies greatly. The result measurement criteria have not been standardized: some papers prioritize predictive performance, others emphasize communication or computational overhead, and still others focus on source or incentive metrics. This heterogeneity has led to different discoveries. Furthermore, there are potential biases because many studies are conducted in a controlled environment with carefully curated datasets, which may not reflect the heterogeneity and noise of real-world medical data. Finally, due to the limited performance index reports and insufficient longitudinal validation, statistical uncertainties (confidence intervals and variability between runs) are rarely reported; large-scale, multi-institutional deployments remain uncommon; and few studies have evaluated long-term stability, scalability under large volumes of clinical data, or regulatory compliance under real-world conditions. These gaps prominently indicate the need for more robust and well-designed research, including prospective clinical trials, to verify the effectiveness, safety, and interoperability of the BCFL framework in actual health care settings.

### Future Prospects

The main obstacles to data sharing and collaboration in the health care system lie in the lack of interoperability and standardization, as well as the absence of standardized benchmark datasets in medical applications in the context of FL and blockchain applications. Therefore, future research must integrate the joint efforts of the government, regulatory authorities, and industry leaders to establish unified technical standards and policy frameworks and give priority to the development of standardized BCFL benchmark datasets. These standards should encompass data formats, transmission protocols, privacy safeguards, and technology implementation guidelines to facilitate seamless data integration and collaboration across diverse health care entities. Standardized datasets should reflect the realistic heterogeneity of medical imaging, EHRs, and multimodal data streams. By providing a common reference point, these benchmarks will enable fair and transparent algorithm comparisons, promote the reproducibility of results, and accelerate the transformation of BCFL innovations into clinical validation tools. In addition, benchmark datasets can be stratified based on disease types, patterns, and clinical tasks (eg, diagnosis, prognosis, and treatment response prediction), thereby allowing for more fine-grained evaluations of system performance in different health care settings. Moreover, regulatory frameworks should prioritize patient privacy and data security, delineate the rights and responsibilities of stakeholders, and foster the compliant adoption of BCFL technology. Comprehensive policy support and technical guidance will be instrumental in mitigating data silos and enhancing the efficiency of collaborative model training.

Scalability and communication efficiency remain two of the most critical technical challenges facing BCFL, particularly in large-scale and high-frequency health care environments. To address the scalability limitations, future research should focus on several key technical optimizations. The first is off-chain computing and side-chain technology [43]. Off-chain computing enables complex training tasks to be executed off-chain, reducing the computational burden on the main blockchain. Meanwhile, side chains can independently handle task-specific transactions, alleviating congestion on the main chain. Layer 2 protocols, such as stateful channels and plasma technology, offer a viable solution by enabling faster transaction processing while preserving blockchain security [43]. These technologies enhance scalability by minimizing main-chain data storage requirements. Another crucial research direction is cross-chain technologies [42]. This approach not only decentralizes workloads and mitigates single-chain bottlenecks but also enhances system-wide parallel processing capabilities. Moreover, cross-chain FL is particularly well-suited for cross-regional and cross-organizational health care collaborations, further strengthening resource management and system security.

Regarding communication cost and efficiency, the following directions are worth exploring in depth. One promising direction is gradient compression. Using gradient compression techniques helps reduce communication overhead. For instance, Konecny et al [127] proposed that structured updates and sketch updates can significantly lower communication costs. However, this approach also introduces potential challenges, such as the loss of relevant information during compression, which may have an impact on the performance of the global model. Therefore, future research should focus on achieving an optimal balance between gradient compression and global model accuracy [128]. Lightweight consensus protocols are also another crucial research direction. Another key technological direction is the Digital Twin [14], which minimizes the need for long-distance data transmission by enabling the generation of virtual models directly on miner nodes. This approach significantly decreases communication latency and costs, making it particularly well-suited for resource-constrained health care environments.

In the field of IoMT, to address the challenges posed by heterogeneous storage, computing, and communication capabilities of medical devices and sensors, future research should prioritize optimizing resource usage. First, lightweight ML models and algorithms, such as model compression and pruning techniques [128], can be developed to alleviate the computational burden on local devices. This not only enhances device operational efficiency but also significantly reduces communication overhead. At the task allocation level, device grouping and hierarchical architectures can be leveraged to allocate computation and data aggregation tasks to devices or intermediate nodes with higher computational capacity, thereby forming a resource-optimized collaborative network.

Another critical aspect is enhancing system robustness in the face of equipment instability. To mitigate this issue, future research should focus on optimizing both failure recovery mechanisms and participant selection strategies. On the one hand, an adaptive connection protocol can be designed to enable devices to automatically rejoin the training process after a connection interruption, ensuring that the global model’s convergence remains unaffected. On the other hand, an optimization strategy based on device availability and performance can be implemented to prioritize the selection of more stable devices for training. Moreover, incorporating a flexible time window would allow devices to complete tasks within a predefined period, thereby enhancing the overall system’s fault tolerance and training efficiency.

Given the highly diverse and statistically heterogeneous nature of medical data in IoMT environments, achieving robust model generalization is another priority. The prevalence of non-IID data across institutions often results in significant discrepancies between global and local models, undermining convergence and performance. Consequently, Wu and Wang [125] have proposed an optimal aggregation algorithm that dynamically adjusts the selection probability of each trainer based on the algorithm’s output. However, trainer selection based on model preferences can severely compromise the generalization capability of the global model. Future research should focus on developing a highly accurate BCFL system with enhanced model generalization. One promising direction is the development of automated data normalization tools, which are capable of recognizing various dataset formats and characteristics, performing automatic transformations to ensure that the data can be directly used by model training [129].

The technical limitations faced by BCFL systems need to be well addressed in the future, and privacy protection is one of the fundamental challenges. Future research should further weigh the relationship between privacy and utility, selecting appropriate privacy-preserving techniques based on system priorities. For instance, in certain scenarios, prioritizing privacy may necessitate the adoption of computationally intensive yet more secure techniques, whereas high-performance requirements may favor more lightweight solutions. Additionally, more advanced privacy-preserving techniques can be explored. For example, zero-knowledge proof (ZKP) is a promising cryptographic method that enables a prover to demonstrate the validity of a statement to a verifier without disclosing any additional information. This ensures that the data owner can validate the accuracy of an update while keeping the original data confidential. This approach not only minimizes the trust overhead among participants but also alleviates the verification burden on clients. Future research should investigate how ZKP can be seamlessly integrated into the BCFL architecture to optimize the balance between performance and privacy, paving the way for a more efficient and secure system with enhanced privacy measures.

Another pressing issue in the development of BCFL systems is the design of fair and dynamic incentive mechanisms to ensure equitable resource allocation among contributors. On the one hand, the Shapley value-based contribution quantification method can be used to assess each participant’s impact on global model performance improvement, thereby enabling a fairer incentive distribution. On the other hand, since variations in data quality directly impact model training effectiveness, data quality-driven incentives can be introduced so that participants contributing high-quality or more representative data receive greater rewards. Moreover, integrating penalty mechanisms is also another crucial research direction [85]. For instance, Cui et al [130] proposed withdrawing tokens when a trainer’s behavior is identified as malicious. Similarly, Weng et al [79] suggested requiring trainers to predeposit tokens, which are forfeited upon detection of malicious activity, but the fairness of this deposit mechanism remains uncertain. Therefore, how to reasonably set the punishment rules under the premise of ensuring fairness still needs further research. These optimization strategies will contribute to building a more effective and equitable BCFL ecosystem, fostering its sustainable development in medical data sharing.

It is undeniable that token-based incentive mechanisms have been widely discussed as a promising approach to encouraging participation in the BCFL network. Although such mechanisms can enhance participation and promote fair resource allocation, they also introduce complex ethical issues. Therefore, in future research, ethical supervision should be embedded in token-based system design, and new solutions should be continuously explored.

Editable mechanisms are expected to be implemented in the near future. Future research could explore how to introduce a moderate degree of editability while ensuring data integrity and security. For instance, the modified Chameleon hash function (also known as the trapdoor hash function) enables controlled modification of blockchain data. When the trapdoor information is available, hash collisions can be efficiently identified, allowing input modifications without altering the hash output. This mechanism facilitates the correction of inaccurate or incomplete data while preserving the structural integrity of the blockchain [8].

In parallel, the interpretability of AI models remains a central concern for the deployment of BCFL in clinical practice. Trust, transparency, and accountability are all closely tied to how well clinicians and patients can understand the rationale behind AI-generated predictions. Existing mainstream interpretability tools (eg, SHAP and LIME) have limitations in the medical field, including difficulties in handling complex distributed data environments and the inability to provide clinically relevant interpretations. Future research should focus on enhancing existing tools and developing interpretability methods adapted to BCFL, such as global-local model comparison techniques, to provide more intuitive and trustworthy interpretations.

In addition to technological and application-level innovations, the future development of BCFL also needs to confront the issue that research mainly remains at the simulation, prototype, and preclinical stages, and these studies have not yet formed a direct correspondence with real medical endpoint indicators. Future work should start from real scenarios: (1) conduct prospective validations in multi-institutional, heterogeneous data and complete clinical workflows to evaluate their actual impact on diagnostic efficiency, therapeutic effect improvement, and resource usage; (2) maintain a balance between security, latency, and maintainability in system design to avoid unacceptable computing and communication costs caused by encryption and on-chain operations; and (3) at the governance level, strengthen the connection with the medical system, regulations and ethical frameworks, and clarify data ownership, responsibility attribution, and auditing mechanisms. Only when technical performance is validated in real clinical settings, system design remains practically deployable, and governance mechanisms are clearly defined, BCFL can progress from laboratory prototypes to clinically reliable infrastructure.

While the above guidance outlines strategic directions for policymakers, clinicians, and implementers, these recommendations remain high-level. To move from broad vision to actionable progress, there is a pressing need for specific evaluative structures that can translate theories into measurable outcomes. Therefore, establishing a good evaluation framework is indispensable, which can provide a foundation for future research, allowing for comparison, verification, and ultimately integration into clinical workflows.

A unified, multidimensional evaluation framework is central to the advancement of BCFL in medicine. From a technical perspective, standardized metrics such as model accuracy, convergence speed, robustness against adversarial attacks, communication latency, and scalability across heterogeneous institutional datasets are indispensable. These indicators establish the baseline scientific validity of BCFL. Equally important afterwards are the clinical assessment criteria, which should reflect the sensitivity and specificity of the diagnosis, its universality in different patient populations and multicenter environments, reduce algorithmic bias, and bring about a tangible improvement in patient prognosis. By embedding clinical endpoints into the assessment, this framework can ensure that technological progress is in line with the real-world health care needs. Finally, there are operational indicators, which should cover interoperability with existing medical data systems, cost-effectiveness, and the long-term sustainability of deployment. By integrating these three dimensions into a unified evaluation system, researchers and clinical workers can establish benchmarks that can both horizontally compare different studies and vertically track research progress. This evaluation framework not only enhances the scientific rigor of BCFL research but also provides an evidence-based basis for decision-making sharing, thereby accelerating the transformation from experimental prototypes to clinical applications.

### Conclusions

This review systematically compiles the research progress of blockchain and FL in the medical field. First, we introduce the theoretical foundations and core technical features of both technologies, analyzing how blockchain enhances the security, privacy protection, and decentralization characteristics of FL, while FL improves the computational efficiency and scalability of blockchain. In addition, we describe the three frameworks and workflows of BCFL. Next, we summarize the research progress in BCFL applications across cross-institutional health care data sharing, the IoMT, public health monitoring, and telemedicine, highlighting its practical value in privacy protection and data collaboration. Moreover, we discuss key challenges in BCFL, including computational efficiency, scalability, data privacy, and incentive mechanism design, while proposing potential solutions and future research directions. The significance of this review lies in providing a comprehensive overview of how BCFL could reshape medical data collaboration and security paradigms, thereby offering valuable insights for researchers and practitioners exploring this interdisciplinary field. However, it is important to acknowledge certain limitations of this review. This review primarily focuses on the theoretical principles and current applications of BCFL, with relatively limited exploration of specific implementation details and performance evaluations. Additionally, most existing BCFL studies rely on simulation experiments or public datasets, lacking validation with large-scale real-world medical data, which affects assessments of its practical feasibility. As such, although BCFL shows promise in supporting future intelligent diagnostics, precision medicine, and collaborative health care systems, claims about clinical readiness remain premature. Future work should focus on prospective, large-scale validation studies, interdisciplinary collaboration with health care providers, and the development of standardized evaluation protocols to ensure that BCFL solutions are clinically safe, ethically sound, and operationally feasible.

## Supplementary material

10.2196/79052 Multimedia Appendix 1 Databases search strategy.

10.2196/79052 Multimedia Appendix 2 Scoring statistics and agreement data.

10.2196/79052 Multimedia Appendix 3 Additional tables.

10.2196/79052 Checklist 1 PRISMA checklist.

10.2196/79052 Checklist 2 Kitchenham SLR Quality Checklist.

## Abbreviations

AI: artificial intelligence
BCFL: blockchain-based federated learning
BFLPD: blockchain-based federated learning for pandemic diagnosis
EHR: electronic health record
EMR: electronic medical record
FBCLC-Rad: Federated Learning–Enabled Blockchain CapsNets Lung Cancer Radiologist
FedAvg: Federated Averaging algorithm
FL: federated learning
FL-BETS: Federated Learning–Based Blockchain-Enabled Task Scheduling
FLED-Block: Federated Learning–Ensembled Deep Learning Blockchain Model
GDPR: General Data Protection Regulation
GRADE: Grading of Recommendations Assessment, Development, and Evaluation
gRPC API: Google Remote Procedure Calls – Application Programming Interface
HIPAA: Health Insurance Portability and Accountability Act
*ICD-10*: *International Statistical Classification of Diseases, Tenth Revision*
*ICD-10-CM*: *International Classification of Diseases, Tenth Revision, Clinical Modification*
IoMT: Internet of Medical Things
IoT: Internet of Things
ML: machine learning
non-IID: non-independent and identically distributed
ODMSM: Optimized Data Management and Secured Federated Learning
PBFT: Practical Byzantine Fault Tolerance
PoS: Proof of Stake
PoW: Proof of Work
PRISMA: Preferred Reporting Items for Systematic Reviews and Meta-Analyses
REST-API: Representational State Transfer – Application Programming Interface
RPC: Remote Procedure Calls
SMPC: Secure Multiparty Computation
ZKP: zero-knowledge proof
