# Correction: Securing Federated Learning With Blockchain in the Medical Field: Systematic Literature Review

**DOI:** 10.2196/95788

**Published:** 2026-04-15

**Authors:** Xudong Wang, Yi Xie, Xiaoliang Chen, Jiaming Yang, Ruiyuan Li, Weihang Gao, Zineng Yan, Hong Zhou, Zhewei Ye

**Affiliations:** 1 Department of Orthopedics, Union Hospital, Tongji Medical College, Huazhong University of Science and Technology, 1277 Jiefang Avenue, Wuhan, Hubei Province, 430022, China, 86 1-397-121-3880; 2 Renmin Hospital of Wuhan University, Wuhan, Hubei Province, China; 3 Department of Orthopedics, People's Hospital of Ningxia Hui Autonomous Region, Ningxia Medical University, Yinchuan, China

 In “Securing Federated Learning With Blockchain in the Medical Field: Systematic Literature Review” [1], the authors noted two errors.

The first affiliation has been changed from the following:

Wuhan Union Hospital, Wuhan, Hubei Province, China

The affiliation now reads:

Department of Orthopedics, Union Hospital, Tongji Medical College, Huazhong University of Science and Technology, Wuhan, Hubei Province, China.

The third affiliation has been changed from the following:

Ningxia Hui Autonomous Region Peoples Hospital, Yinchuan, Ningxia, China.

The affiliation now reads:

Department of Orthopedics, People's Hospital of Ningxia Hui Autonomous Region, Ningxia Medical University, Yinchuan, China.

The corrections will appear in the online version of the paper on the JMIR Publications website, together with the publication of this correction notice. Because these were made after submission to PubMed, PubMed Central, and other full-text repositories, the corrected article has also been resubmitted to those repositories.
