# Supplementary material for: Securing Federated Learning With Blockchain in the Medical Field: Systematic Literature Review
Source: J Med Internet Res. 2026 Feb 19;28:e79052. doi: 10.2196/79052 (PMC12919988; doi:10.2196/79052)
Supplement: Multimedia Appendix 1 [file jmir-v28-e79052-s001.docx]

# Databases Search Strategy

To ensure the comprehensiveness and timeliness of this review, the literature data mainly come from four well-known academic databases, including PubMed, IEEE Xplore, Web of Science and Google Scholar. The time range is mainly set from January 2018 to February 2025. It also incorporates some early pioneering studies to trace the theoretical development and technological evolution of BCFL in the medical field. In this search strategy, PubMed is the core source for obtaining authoritative literature on the biomedical and clinical applications of this technology. IEEE Xplore provides key technical details and algorithmic innovations in the field of computer science and engineering. Web of Science ensures the academic quality of the selected literature and helps us trace the research context with its strict citation index. Google Scholar, as an important supplement, captures the latest achievements with its extensive coverage, minimizing the omission of literature to the greatest extent.

Review search strings used for each database

| PubMed | (("blockchain"[Title/Abstract] OR "distributed ledger"[Title/Abstract] OR "distributed ledger technology"[Title/Abstract] OR "DLT"[Title/Abstract] OR "blockchain"[MeSH Terms])  AND  ("federated learning"[Title/Abstract] OR "federated* learn*"[Title/Abstract] OR "collaborative learning"[Title/Abstract] OR "distributed machine learning"[Title/Abstract] OR "federated learning"[MeSH Terms])  AND  ("healthcare"[Title/Abstract] OR "health care"[Title/Abstract] OR "medical"[Title/Abstract] OR "clinical"[Title/Abstract] OR "electronic health record"[Title/Abstract] OR "electronic medical record"[Title/Abstract] OR "EMR"[Title/Abstract] OR "IoMT"[Title/Abstract] OR "telemedicine"[Title/Abstract] OR "epidemic"[Title/Abstract] OR "COVID-19"[Title/Abstract] OR "public health"[Title/Abstract]))  AND ("2018/01/01"[PDAT] : "2025/02/15"[PDAT])  AND (english[Filter]) |
| --- | --- |
| IEEE Xplore | ((("Document Title":"blockchain") OR ("Abstract":"blockchain") OR ("Index Terms":"blockchain") OR ("Document Title":"distributed ledger") OR ("Abstract":"distributed ledger"))  AND  (("Document Title":"federated learning") OR ("Abstract":"federated learning") OR ("Index Terms":"federated learning") OR ("Abstract":"collaborative learning") OR ("Abstract":"distributed machine learning" ))  AND  (("Abstract":"healthcare") OR ("Abstract":"medical") OR ("Abstract":"clinical") OR ("Abstract":"IoMT") OR ("Abstract":"telemedicine") OR ("Abstract":"electronic health record" ))  )  AND PublicationYear:2018-2025 |
| Web of Science | TS=(("blockchain" OR "distributed ledger" OR "distributed ledger technology" OR "DLT")  AND ("federated learning" OR "federated* learn*" OR "collaborative learning" OR "distributed machine learning")  AND ("healthcare" OR "health care" OR "medical" OR "clinical" OR "electronic medical record" OR "EMR" OR "IoMT" OR "telemedicine" OR "public health" OR "COVID-19"))  AND PY=(2018-2025) |
| Google Scholar | "blockchain" AND ("federated learning" OR "federated learn*" OR "collaborative learning" OR "distributed machine learning") AND (healthcare OR medical OR clinical OR EMR OR IoMT OR telemedicine OR "public health" OR COVID-19) |

# Databases Inclusion and Exclusion Criteria

| Criteria | Inclusion Criteria | Exclusion Criteria |
| --- | --- | --- |
| Language | English | Non-English |
| Keywords | Includes terms like "Blockchain," And "federated learning," And "Healthcare" | There are no such keywords as "healthcare", "medicine", "clinical", "electronic medical records", "epidemic", "Internet of Things healthcare" or "telemedicine" |
| Article type | Review, peer-reviewed journal articles, theoretical models, as well as papers from various top journals and top-level conferences | Conference abstracts, non–peer-reviewed articles, and editorials and opinion articles lacking technical details or empirical verification. |
| Research Focus | Studies focusing on the theoretical framework, system architecture, empirical evaluation or case study of BCFL | Studies focusing on blockchain and federated learning, but does not involve studies on medical applications. |

# Databases Search Strategy

| Step | Description | PubMed | IEEE Xplore | Web of Science | Google Scholar | Total |
| --- | --- | --- | --- | --- | --- | --- |
| Raw Keyword Search | Articles identified through database searches. Keywords: Blockchain, federated learning, Healthcare, etc. | 288 | 465 | 624 | 1170 | 2547 |
| Duplicates and Non-English Removed | Clear non-English literature and then use EndNote software to remove duplicate literature records. | | | | | 1327 |
| Title/Abstract Screening | Articles screened for relevance based on title/abstract. | 319 | | | | |
| Focused Screening | Articles focusing on the theoretical framework, system architecture, empirical evaluation or case study of BCFL. | 111 | | | | |
| Additional Studies | Additional relevant studies identified from references lists. | 19 | | | | |
| Total Studies Included | Final number of studies included in the review. | 130 | | | | |
