# Supplementary material for: Securing Federated Learning With Blockchain in the Medical Field: Systematic Literature Review
Source: J Med Internet Res. 2026 Feb 19;28:e79052. doi: 10.2196/79052 (PMC12919988; doi:10.2196/79052)
Supplement: Multimedia Appendix 3 [file jmir-v28-e79052-s003.docx]

Table. 1. Comparison of Technical Characteristics: Blockchain vs. Federated Learning

| Technical Characteristic | Blockchain | ᵃFL | Advantages of Integration |
| --- | --- | --- | --- |
| Data Storage Mode | Decentralized ledger | Local storage | Eliminates single points of failure and enhances data security by reducing privacy risks. |
| Security | Ensures data integrity through immutability, cryptographic hash functions, and consensus mechanisms. | Protects data via encryption, differential privacy, and secure gradient transmission， etc. | Establishes a dual-layer security framework, mitigating risks of data tampering and privacy breaches. |
| Trust Mechanism | Achieves trustless transactions through decentralization and smart contracts. | Stores client data locally and only shares model parameters. | Blockchain-based consensus and smart contracts enhance trust, enabling a fully decentralized FL framework. |
| Cross-Institutional Collaboration | Suitable for multi-party data maintenance but lacks efficient data processing capabilities. | Enables institutional cooperation but relies on a trusted central aggregator. | Smart contracts automate data-sharing agreements, improving collaboration efficiency. |
| Auditability & Regulatory Compliance | Transaction logs ensure traceability, supporting compliance with regulations such as GDPR and HIPAA. | Patient data is stored locally and only model parameters are shared, resulting in often opaque training processes. | Blockchain-based audit trails, combined with FL’s privacy-preserving techniques, enhance transparency and compliance. |

ᵃFL：Federated Learning

Table. 2. Blockchain’s Role in Addressing Federated Learning Challenges

| Challenges in Federated Learning | Blockchain-Based Solutions | Specific Mechanisms |
| --- | --- | --- |
| Single Point of Failure | Decentralized ledger | Utilizes a consensus mechanism to ensure data consistency and fault tolerance in distributed networks. |
| Lack of Trust Among Participants | Immutability and transparency | Implements time-stamped logging and on-chain auditing to enhance data traceability and trust. |
| Model Tampering and Poisoning Attacks | Immutability | Employs cryptographic hash functions, digital signatures, and smart contracts to verify model updates and filter out malicious submissions. |
| Lack of Effective Incentive Mechanisms | Smart contracts and token incentives | Distributes rewards based on contribution quality through token incentives and reputation scoring mechanisms. |
| Excessive Communication Overhead | Distributed storage | Reduces communication burden by integrating off-chain storage with on-chain hash verification. |
| Scalability Constraints | Blockchain scalability solutions | Leverages off-chain computation and sidechain technologies to alleviate computational bottlenecks. |
