# Supplementary material for: Securing Federated Learning With Blockchain in the Medical Field: Systematic Literature Review
Source: J Med Internet Res. 2026 Feb 19;28:e79052. doi: 10.2196/79052 (PMC12919988; doi:10.2196/79052)
Supplement: Checklist 1 [file jmir-v28-e79052-s004.docx]

**PRISMA (Preferred Reporting Items for Systematic Reviews and Meta-Analyses) Checklist**

| **PRISMA-IPD**  **Section/topic** | **Item No** | **Checklist item** | **Reported on page** |
| --- | --- | --- | --- |
| **Title** | | | |
| Title | 1 | Securing federated learning with blockchain in medical field: a systematic literature review | 1 |
| **Abstract** | | | |
| Structured summary | 2 | Provide a structured summary including as applicable: | 1 |
|  |  | **Background**: With the development of intelligent healthcare driven by medical data, it is a major challenge to share sensitive medical data safely and effectively among institutions. This study aims to comprehensively review the latest progress of blockchain-based federated learning (BCFL) in the medical field. By exploring the collaborative integration of federated learning and blockchain, this paper assesses how BCFL enhances data security, supports cross-institutional collaboration for privacy protection, and promotes practical applications in the healthcare sector. |  |
|  |  | **Methods**: The literature data mainly come from PubMed, IEEE explore, Web of Science and Google Scholar. Meanwhile, focusing on the literature from 2018 to the present, some early pioneering studies were also included to trace the theoretical development and technological evolution of BCFL in the medical field. The inclusion criteria include: (1) Research involving blockchain and federated learning technologies and exploring their medical applications; (2) The types of literature cover high-quality journal articles, international conference papers and authoritative reviews to ensure the reliability of the research; Exclusion criteria include: (1) Research that only focuses on blockchain or federated learning without medical applications; (2) Repeated or highly similar studies. |  |
|  |  | **Results**: Search for studies from 2018 to 2025 using Boolean logic and domain-specific keywords. And after automatic deduplication and multi-stage manual screening, more than 100 high-quality papers were selected. These works cover the theoretical research, technical architecture, application scenarios, challenges and future trends of BCFL. These studies provide a solid academic foundation for in-depth exploration of the application of BCFL in medicine. |  |
|  |  | **Discussion:** Blockchain-based federated learning represents a transformative paradigm of secure, collaborative and privacy-protecting medical artificial intelligence, demonstrating strong potential in supporting precision medicine, global health data collaboration and large-scale artificial intelligence deployment in the healthcare sector. However, BCFL also has problems such as scalability, communication cost, incomplete incentive mechanism and insufficient interpretability. |  |
|  |  | **Other:** None. |  |
| **Introduction** | | | |
| Rationale | 3 | The exponential growth of medical data and the advancement of artificial intelligence have accelerated the development of data-driven healthcare. However, due to privacy issues, data silos and regulatory restrictions, the secure and effective sharing of sensitive medical data among various medical institutions remains a major challenge. Traditional centralized systems are prone to data leakage and single point of failure, while existing privacy protection technologies are confronted with high computing and communication costs. This article explores the combination of federated learning and blockchain, two privacy protection technologies, to establish a medical system that promotes trust, security and data integrity. | 1-3 |
| Objectives | 4 | This study aims to comprehensively review the latest progress of blockchain-based federated learning (BCFL) in the medical field. By exploring the collaborative integration of federated learning and blockchain, this paper assesses how BCFL enhances data security, supports privacy-preserving cross-institutional collaboration, and promotes practical applications in the healthcare sector, including medical data sharing, IoMT, public health monitoring, and telemedicine. | 1 |
| **Methods** | | | |
| Protocol and registration | 5 | Not published in other journals |  |
| Eligibility criteria | 6 | To ensure relevance and academic rigor, this review has established strict inclusion and exclusion criteria. The inclusion criteria include: The inclusion criteria include (1) the studies must involve blockchain and federated learning technologies and explore their medical applications; (2) the kiterature published in peer-reviewed journals and reviews indexed in SCI/SSCI or in top-tier international conferences (e.g., IEEE, ACM). Seminal papers with >30 citations were included regardless of publication venue.; and (3) reported theoretical frameworks, system architectures, empirical evaluations, or case studies The exclusion criteria then include (1) studies focusing solely on blockchain or federated learning without medical applications; (2) editorial and opinion articles that lacking technical details or empirical validation; and (3) duplicate reports or low-quality publications from non-peer-reviewed. | 6 |
| Identifying studies - information sources | 7 | The literature data was primarily retrieved from several prominent academic databases, including PubMed, IEEE Xplore, Web of Science, and Google Scholar. To ensure the timeliness of the research, the review focuses on literature published between January 2018 and February 2025, while also incorporating some early seminal studies to trace the theoretical development and technological evolution of BCFL in the medical field. | 5 |
| Identifying studies - search | 8 | The search strategy employs Boolean logic operators to formulate a comprehensive formula, e.g., ("blockchain") AND ("federated learning") AND ("medical" OR "healthcare"). To enhance retrieval efficiency and encompass interdisciplinary intersections, the search terms are further expanded, such as "(Blockchain-enabled Federated Learning)" "(Distributed Machine Learning)""(Decentralization)" "(Internet of Medical Things (IoMT)" "(Telemedicine)" "(EMR)" "(Epidemics)" are incorporated to ensure literature comprehensiveness. The exact search string is as follows: ("blockchain" OR "distributed ledger technology") AND ("federated learning" OR "collaborative learning" OR "distributed machine learning") AND ("healthcare" OR "medical" OR "clinical" OR "EMR" OR "(Epidemics)" OR "IoMT" OR "telemedicine"). | 5-6 |
| Study selection processes | 9 | According to the search strategy, 2,547 documents were initially retrieved. After automatic deduplication by EndNote, 1,327 were retained. Subsequently, two independent reviewers (Reviewer A and Reviewer B) manually screened all the literature in two stages based on the inclusion and exclusion criteria. Firstly, based on the title and abstract, select high-quality reviews, papers from top journals and conferences, and literature that can clearly explain the application of BCFL in the healthcare field, totaling over 300 articles. In the second stage, further review was conducted. Through full-text reading, studies lacking empirical verification, technical details or experimental data were eliminated, and ultimately over 100 high-quality documents were retained. In addition, to ensure the comprehensiveness of the literature, this review also referred to the latest review papers and included the core research results cited therein to avoid missing key progress. | 6-7 |
| Data collection processes | 10 | Extract the topics, fields, datasets, performance metrics and limitations involved in the article. For the content not mentioned, collect it from the supplementary documents of the article. If necessary, send an email to the author's email address to inquire about the relevant data. | 46-54 |
| Data items | 11 | Extract from the references the involved topics, fields, the BCFL framework used, the datasets used, the purpose of the application, the final performance indicators (such as accuracy rate, ROC curve, interception rate of malicious attacks), the limitations of the framework and the future development trends. | 46-54 |
| Risk of bias assessment in individual studies. | 12 | After the screening is completed, the reviewers evaluate the quality of the literature. Since the corpus of this review mainly comes from the cross-disciplinary research of medicine, engineering and computer science, the GRADE framework commonly used in clinical evidence-based studies is not applicable. We used the SLR quality checklist based on the Kitchenham principle[18] to score each of the 12 indicators of the included studies, including reproducibility, method transparency, evaluation design, data description, experimental validity, attack/privacy discussion, and reproducibility, item by item (1/0.5/0). Two independent reviewers will assess each document. If there are significant differences in the scores (the total score difference of a single document is greater than 2 or there are differences in key items), a third senior reviewer will arbitrate. Ultimately, the studies were classified into three quality grades based on the total score: high (≥9), medium (5-8), and low (<5). When writing summaries and conclusions, give priority to citing high-quality research. |  |
| Specification of outcomes and effect measures | 13 | Compare the medical effect indicators (AUC, sensitivity, accuracy rate, ROC, etc.) in different medical scenarios, compare the malicious attack interception rate and privacy protection efficiency under different BCFL frameworks, and compare the communication overhead and communication efficiency of different frameworks. |  |
| Synthesis methods | 14 | By listing tables to present the performance indicators of different BCFL frameworks in various medical scenarios, the superiority of the BCFL framework in the development of modern healthcare is demonstrated. Demonstrate through schematic diagrams how the BCFL framework participates in different medical scenarios. At the same time, list the datasets used by the author to reflect the interpretability of the article. Meanwhile, a hierarchical evidence table was listed to correspond the BCFL architecture innovation with clinical outcomes, deployment environments, and validation depth, revealing the bottlenecks in the current BCFL process from conceptual framework to clinical validation, and providing a directional reference for future research design. Finally, the limitations and shortcomings of the article are also written to provide inspiration for future research. | 46-54 |
| Risk of bias across studies | 15 | This study conducted cross-paper comparisons of similar experimental indicators and marked the highly biased articles. At the same time, for references lacking key indicators, contact the corresponding author to obtain them. If there is no response, mark the evidence gap. Mark highly biased articles that exaggerate medical value in the references (such as claiming "to support real-time ICU monitoring"). |  |
| Additional analyses | 16 | None. |  |
| **Results** | | | |
| Study selection | 17 | We conducted a systematic literature review using four databases. Search for research from January 2018 to February 2025 using Boolean logic and domain-specific keywords. Through the above search strategy, 2,547 documents were initially retrieved. Subsequently, duplicate references were automatically deleted through the EndNote software, and 1,327 references were retained. Meanwhile, to ensure relevance and academic rigor, this review established strict inclusion and exclusion criteria. This review went through a two-stage manual screening process: Firstly, 319 articles were screened based on titles and abstracts to retain those explicitly related to BCFL and its medical applications. In the next stage, a full-text review will be conducted to eliminate research lacking empirical verification, theoretical rigor or experimental data. In addition, the review also referred to the latest review papers and included the core research results cited therein. Ultimately, over 100 high-quality papers will be retained. | 5-7 |
| Study characteristics | 18 | For each literature, this review extracted the medical application scenarios of the BCFL framework, the datasets used, the final performance indicators, the limitations faced, and the future development trends, and presented these data in a table. | 46-54 |
| Risk of bias within studies | 19 | The article mainly summarizes the application of the BCFL framework in medical scenarios and does not conduct bias risk analysis on specific data. |  |
| Results of individual studies | 20 | We used the SLR quality checklist based on the Kitchenham principle to score each of the 12 indicators of the included studies one by one, including reproducibility, method transparency, evaluation design, data description, experimental validity, attack/privacy discussion, and reproducibility. Ultimately, they are classified into three categories based on their scores:  • ≥9 (High quality) : It is given priority for citation and used as key evidence in comprehensive argumentation and evidence presentation, and has higher explanatory power. Experimental papers include well-designed randomized controlled trials or high-quality quasi-clinical trials with minimal risk of bias, consistent research results, direct relevance to the review questions, and precise effect estimation, that is, multi-center trials with repeatable results in a medical data environment or robust blockchain joint learning experiments.  • 5-8 (moderate) : Reserved for supportive discussions or as exploratory evidence. Such studies typically involve smaller clinical data validation or heterogeneous patient cohorts, but still provide findings that are generally supportive...  • <5 (Low quality) : Use with caution, or only as background information and to emphasize trends. The evidence mainly comes from partial preprints, or observational studies with significant methodological limitations, single-center pilot studies, or reports with serious biases, inconsistencies or indirect risks. | 46-54 |
| Results of syntheses | 21 | Most of the papers are engineering-centered: the main research designs are system prototypes, simulation studies and preclinical studies, which propose and evaluate the BCFL architecture under various privacy threats or resource integrations. Common data types include clinical records and diagnostic images (including public and institutional-specific datasets), while some work on the medical Internet of Things assesses content such as sensor flows. The evaluation metrics for the report vary by procedure, but typically include predictive performance (accuracy, sensitivity, AUROC), communication and computational overhead, privacy/leakage metrics, as well as auditability or source metrics. |  |
| Risk of bias across studies | 22 | To address the limitations of treating all included studies equally, we adopted a weighted approach based on study design and methodological quality. High-quality research is given greater explanatory weight; Moderate evidence serves as supportive or exploratory evidence; The results of low-quality studies, however, are used with caution or merely as background information and to emphasize trends. This approach ensures that the conclusions of the review reflect the overall strength of the existing best evidence and reduces the possibility of misleading interpretations caused by low-quality research. |  |
| Additional analyses | 23 | None. |  |
| **Discussion** | | | |
| Summary of evidence | 24 | This review demonstrates the superiority of combining the decentralized trust and auditability of blockchain with the privacy-protecting collaborative learning capabilities of FL. This integration reduces risks such as model tampering, data leakage, and lack of incentives in federated systems. Subsequently, different BCFL frameworks were presented, including architectures of fully coupled, flexibly coupled and loosely coupled models, providing different trade-offs among efficiency, scalability and security respectively. And it describes the application of the BCFL framework in different healthcare fields at the present stage, including cross-institutional medical data sharing, medical Internet of Things (IoMT), epidemic prediction and telemedicine. | 7-55 |
| Limitations | 25 | The current limitations of the BCFL framework are expounded, including application types: insufficient system interoperability and scalability, excessively high communication costs, stability issues of hardware devices, and heterogeneity of medical data. Technical aspects: privacy and security issues, imperfect incentive mechanisms, insufficient editable ability, and insufficient model interpretability. Although the studies included in this review collectively provide valuable insights into the potential of blockchain-enabled federated learning (BCFL) in healthcare, several limitations must be acknowledged. First, many of the available studies are conceptual frameworks, simulations, or small-scale case studies rather than large-scale clinical implementations. This reliance on theoretical or laboratory-based evidence introduces a risk of overestimating feasibility and underestimating real-world deployment challenges. Second, there is a degree of heterogeneity among the included studies in terms of data sources, evaluation metrics, and implementation settings. Such variability makes direct comparison difficult and limits the ability to draw firm conclusions about generalizability. In addition, potential biases exist because many studies are conducted in controlled environments with carefully curated datasets, which may not reflect the heterogeneity and noise of real-world medical data. Finally, several key results remain imprecise due to limited reporting of performance metrics and insufficient longitudinal validation. Few studies evaluate long-term stability, scalability under high-volume clinical data streams, or regulatory compliance in real-world conditions. | 55-59 |
| Conclusions | 26 | The significance of this review lies in providing a comprehensive perspective on the integration of blockchain and FL, summarizing existing research achievements and challenges, and offering valuable insights for the academic and industrial sectors to promote the further development of BCFL in the healthcare field. Overall, BCFL holds significant implications for advancing precision medicine, accelerating multi-institutional research, and fostering global data collaboration. By reducing data silos and enabling trustworthy analytics, BCFL could support earlier disease detection, more personalized treatment strategies, and greater healthcare equity. Future work should focus on large-scale clinical validation and regulatory alignment to ensure BCFL systems are safe, effective, and deployable in real-world healthcare environments. | 64-65 |
| Funding | | | |
| Funding | 27 | This research was funded by the National Natural Science Foundation of China (Project Nos. : 81974355, 82172524), the Key Research and Development Program of Hubei Province (Project No. : 2021BEA161), the National Innovation Platform Cultivation Program (Project No. : 2020021105012440), and the Education Reform Project of Ningxia Medical University (Project No. : Funded by NYJY2025057. | 65 |
