# Supplementary material for: Securing Federated Learning With Blockchain in the Medical Field: Systematic Literature Review
Source: J Med Internet Res. 2026 Feb 19;28:e79052. doi: 10.2196/79052 (PMC12919988; doi:10.2196/79052)
Supplement: Checklist 2 [file jmir-v28-e79052-s005.docx]

## Kitchenham SLR Quality Checklist

This is an SLR quality assessment form (including scoring rules, scoring criteria, judgment thresholds and review processes) based on Kitchenham's approach and designed for the application of the blockchain-based federated Learning Framework (BCFL) in the healthcare field, which is compatible with engineering/computer science papers.

I. Scoring rules :

• 1 = criterion fully met

• 0.5 = Partially met / Defective

• 0 = Not met / No relevant information found

## II. Quality Assessment Standard Table

| # | Quality Item | Assessment criteria |
| --- | --- | --- |
| 1 | Clarity of research aims and questions. | Clearly stating the research objectives or questions, hypotheses, or research questions (RQ) helps determine whether the research purpose aligns with the topic/conclusion. |
| 2 | Background and Context Description (Context & Motivation) | Describe the research background, application scenarios, or data sources (such as EHR, IoMT equipment, image data, etc.), and explain why this issue is important. |
| 3 | Related work and contributions | Does this paper compare with existing work, and clearly indicate its contributions (theory/method/system/experiment) and differences? |
| 4 | Methodological transparency in method/architecture description | Are the algorithm, system architecture, protocol, and process description sufficient for human understanding and reproduction (pseudocode, flowchart, module description)? |
| 5 | Experimental design / Evaluation | The evaluation metrics, baseline comparisons, dataset partitioning, cross-validation, and hyperparameter selections should be appropriate and clear. |
| 6 | Data and data processing transparency (Data availability & preprocessing) | Are the data source, scale, distribution, preprocessing, and privacy processing (de-identification/simulation/synthesis) clearly reported? Is the data disclosed or is the reason for not disclosing it explained? |
| 7 | Security & privacy threat analysis | Does it analyze potential attack surfaces (data breaches, model poisoning , reverse engineering, etc.) and provide countermeasures or discuss limitations? |
| 8 | Performance/Scalability/Overhead Assessment | Whether to report time, communication, and computational overhead; performance under different numbers of nodes/data heterogeneity. |
| 9 | Validity and statistical significance (Results validity) | Whether the reported results have undergone statistical testing, confidence intervals, or multiple experiments to demonstrate stability. |
| 10 | Implementation and Reproducibility | Does it provide code repository, model weights, configuration, or detailed implementation information to support reproduction? |
| 11 | Discussion and Limitations (Threats to validity / Limitations) | Does it explicitly discuss internal/external/structural validity threats and research limitations? |
| 12 | Conclusion consistency with evidence | Whether the conclusion is supported by the results, and whether there are exaggerated or inconsistent statements with the evidence. |

III. Scoring Strategies and Processes

1. Item-by-item scoring: Two independent reviewers used the table above to score each article item by item (0/0.5/1).

2. Total score calculation: Add up the 12 items, the total score ranges from 0 to 12.

3. Threshold recommendations (can be adjusted as needed):

• ≥9 (High Quality): Highly cited and considered key evidence in comprehensive argumentation and presentation , possessing greater explanatory power. (Experimental papers include well-designed randomized controlled trials or high-quality quasi-clinical trials with minimal risk of bias, consistent research results, direct relevance to the review questions, and precise effect estimation; that is, multi-center trials with repeatable results in the medical data environment or robust blockchain joint learning experiments . )

• 5–8 (Moderate): Reserved for supporting discussion or as exploratory evidence . ( Such studies typically involve smaller-scale clinical data validation or heterogeneous patient cohorts, but still provide generally supportive findings. )

• <5 (Low Quality): Use with caution, or only as background information and to emphasize trends. ( The evidence mainly comes from partial preprints, or observational studies with significant methodological limitations, single-center pilot studies, or reports with serious bias, inconsistency, or indirect risks. )

• It should be noted that for review articles, the Kitchenham quality assessment methods cited, and the corresponding laws and regulations, quality items 4 to 9 can be ignored. Meanwhile, the total scores of items 1 to 3 and items 10 to 12 are raised to 2 points.

4. Arbitration Mechanism: If the difference between two reviewers in the total score or key items (such as methodological completeness, data transparency) of a certain article is greater than 2 points, or if there is a disagreement on a single item, the article shall be submitted to a third senior reviewer for arbitration.

⸻
